# Hypershape Recognition: A General Framework for Moment-Based Molecular Similarity

**DOI:** 10.1021/acs.jcim.5c00555

**Published:** 2025-06-11

**Authors:** Marcello Costamagna, Marco Foscato, David Grellscheid, Vidar R. Jensen

**Affiliations:** † Department of Chemistry, 1658 University of Bergen, Allégaten 41, Bergen N-5007, Norway; ‡ Department of Informatics, 1658 University of Bergen, Thormøhlensgate 55b, Bergen N-5006, Norway

## Abstract

Due to the widespread use of molecular similarity assessments in drug design, numerous methods for the calculation of similarity scores of organic molecules have been developed. When applied to other types of molecules, such as inorganic and organometallic compounds, these methods face significant challenges. To overcome these challenges, we here introduce Hypershape Recognition (HSR), a versatile framework for moment-based similarity assessment of three-dimensional (3D) chemical representations annotated with atomic features. In a default, general-purpose, implementation of the framework, features containing information about the atomic number, the isotope (the number of neutrons), and the formal charge of each atom are combined with its Cartesian coordinates to form the *N*-dimensional objects, termed *hypershapes*, that are compared. The *hypershapes* may account for any atomic features, including, as the first moment-based similarity method, any user-provided numerical values. Thus, the HSR framework can be tailored for specific applications, such as that of distinguishing between isotopologues and transition-metal complexes with different oxidation states, not handled by other moment-based molecular similarity methods. Moreover, by placing each *hypershape* in a reference system consisting of its own principal components (PCs, derived from principal component analysis, PCA, of the centered *N*-dimensional coordinates and features of the *hypershape*) and using reference points located on PCs instead of on atoms to generate distance distributions and their moments, HSR similarity scores are continuous across geometry fluctuations. The PC-based reference system also enables HSR to distinguish between enantiomers. HSR is available as open source at https://github.com/denoptim-project/HSR.

## Graphical abstract

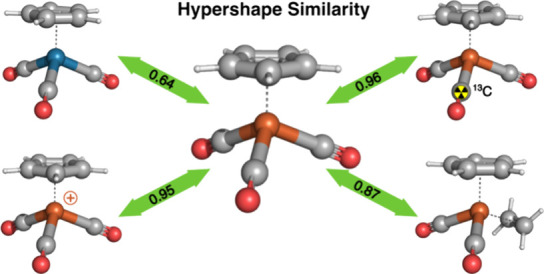

## Introduction

Chemical entities, such as materials and molecules, of similar structure tend to have similar properties.^1–3^ This principle, sometimes referred to as the Similarity Property Principle (SPP),^4^ is a cornerstone in cheminformatics methods and applications, spanning drug design,^5–7^ metabolomics,^8^ and material science.^9,10^ In fact, at least in a first approximation, chemical entities can be efficiently searched, ranked, and classified according to their expected properties using easy-to-compute similarity measures rather than by predicting their properties via costly physics-based computations.

In general, any definition of a similarity measure relies on a representation of the chemical entities being compared. Next, such representations are either compared directly^11^ or, more commonly, used to compute fingerprints (i.e., fixed-size vectors that condense the features of chemical entities),^12^ which are then compared by algorithms that calculate a numerical degree of similarity.^12–14^

Historically, chemical similarity methods have relied heavily on topological representations, such as those of strings, as in the Simplified Molecular Input Line Entry System (SMILES), and graphs.^15^ Their simplicity, intuitiveness, and computational efficiency,^16^ make such representations attractive for similarity evaluation over large libraries of organic compounds.^17^ In many cases, especially for virtual screening of organic compounds, similarity measures based on such representations have proven sufficient and sometimes even superior to those based on 3D representation.^18,19^

Despite their efficiency, string- and graph-based representations do not accurately capture 3D information and bonding interactions beyond those handled by standard valence rules.^20^ String- and graph-based representations as well as fingerprints derived thereof^12^ are thus less suited to identify and compare chemical entities with features beyond those of organic compounds, such as organometallic complexes.^21^

These limitations hamper the development and use of cheminformatics methods far beyond classical inorganic chemistry, and even affect medicinal chemistry,^21,22^ with metallodrugs and radioactive tracers for medical imaging being but some of the compound classes for which string- and graph-based representations are frequently unsuitable.^23–25^ The need for technology capable of handling and manipulating chemical and structural information on broad ranges of compounds^26,27^ is exacerbated by the emergence of methods for virtual screening and de novo design of organometallic compounds catalysts^28–30^ and materials.^31–33^ The latter methods do not appear to have been used in conjunction with molecular similarity metrics,^21^ illustrating the challenges that have retarded the development of methods for the analysis and comparison of inorganic and organometallic compounds. Despite ongoing efforts to extend the applicability of topological representations,^34,35^ there are currently no standardized strategies ensuring unambiguous representation of features particular to, for example, transition-metal and organometallic compounds, such as some kinds of stereochemistry and valence-rules-violating bonding interactions.^20,36^ Hence, when applied to such compounds, similarity methods based on standard topological representations suffer from inconsistencies in their use of various work-arounds.

Fortunately, the inconsistencies can, with some limitations,^20^ be eliminated by using 3D representations, which are thus the focus of the present work. The 3D similarity methods can be divided into two main categories: superposition-based methods and moment-based methods.^2,37,38^ An alternative approach, the SOAP (Smooth Overlay of Atomic Positions) descriptor,^39^ can be also used in similarity comparisons. This descriptor, however, has primarily been used in materials design^40^ and does not accommodate features beyond the atomic number of each atom. SOAP has therefore not been considered further in this work.

Turning now to superposition-based methods, these involve alignment of the 3D structures to maximize their overlap.^41^ Methods such as ROCS (Rapid Overlay of Chemical Structures)^11^ offer direct comparison of molecular shapes along with the possibility for visual inspection. While this approach is effective,^42,43^ it remains unclear whether it is inherently more accurate than alignment-free alternatives.^44^ Furthermore, the alignment comes with a potentially high computational cost.^45^

In contrast, moment-based methods are based on fingerprints that are much faster to handle computationally. The fingerprints are produced from numerical descriptors that, together, approximate the original 3D shape.^45^ The speed with which such fingerprints can be processes makes them suitable for analyzing and screening large libraries of compounds, as demonstrated in drug-design applications.^46,47^

The Ultrafast Shape Recognition (USR) method^47–49^ is the prime and first example of such fast, moment-based similarity measures^50^ and it has been used in several prospective studies.^44,51–54^ USR captures the 3D shape of a molecule in a fixed-length fingerprint of 12 numbers (Figure 1). These 12 numbers consists of the first three statistical moments (i.e., the mean, the standard deviation, and the skewness) of the distributions of distances between each atom and the following list of reference points: (i) the molecular centroid (point labeled *ctd* in Figure 1), i.e., the average of the coordinates of all the atoms in the molecule, (ii) the atom closest to the centroid (labeled *ctc*), (iii) the atom farthest from the centroid (labeled *ftc*), and (iv) the atom farthest from *ftc* (labeled *ftf*).

**Figure 1:**
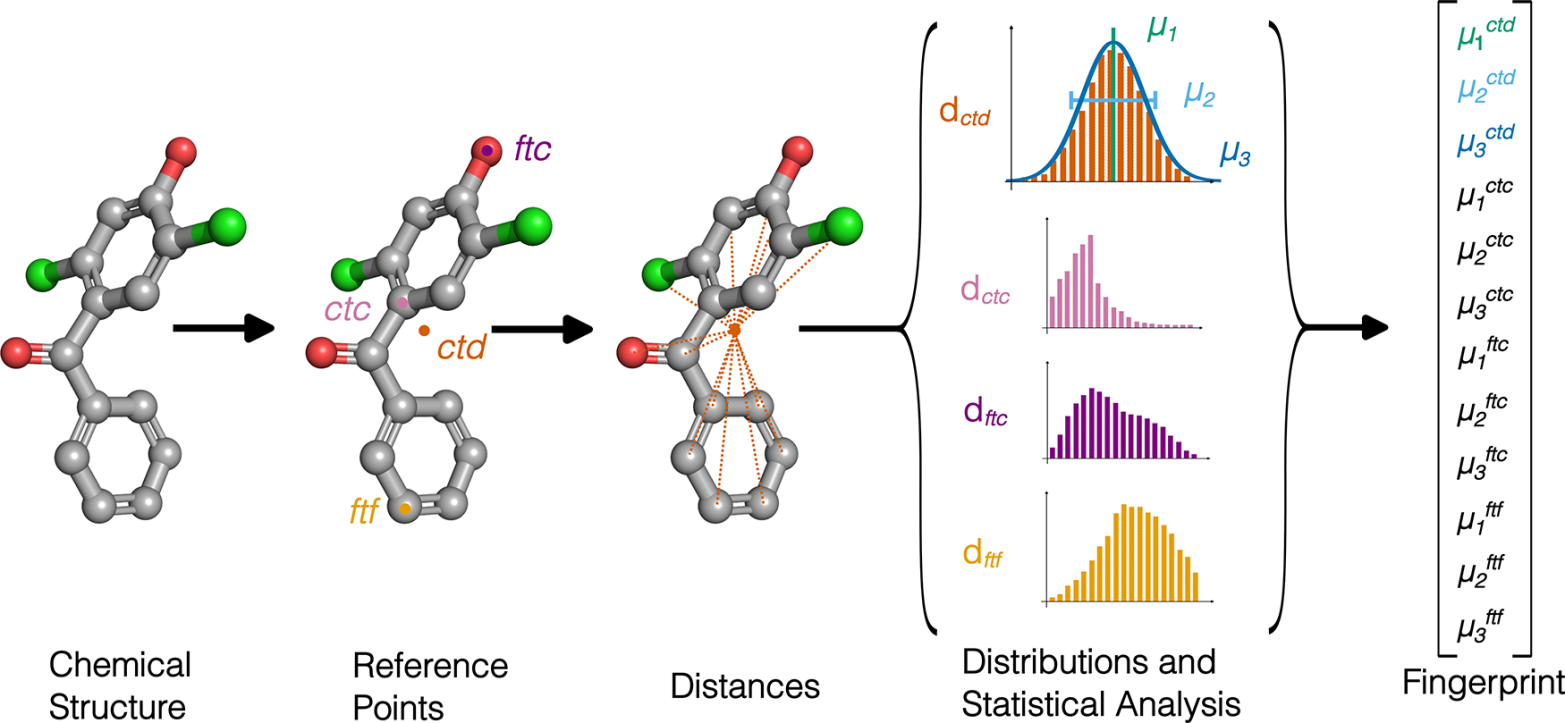
Generation of the fingerprints of the Ultrafast Shape Recognition (USR) method. From left to right: Given a molecular structure, four reference points are identified: the geometrical center of the molecule (*ctd*), the atom closest to *ctd* (*ctc*), the atom farthest from *ctd* (*ftc*), and the atom farthest from *ftc* (*ftf*). Then, the distances between each atom and each reference point are collected into four distributions (d*_ctd_*, d*_ctc_*, d*_ftc_*, d*_ftf_*). The first three statistical moments, i.e., the mean (μ_1_), the standard deviation (μ_2_), and the skewness (μ_3_), of each distribution collectively define the fingerprint.

Since the 12 moments describe the main features of the four distance distributions, they effectively compress 3D information into a fixed-size fingerprint, capturing the overall shape of the point cloud as viewed from the reference points. Given a query (***q***) and a target (***t***) fingerprint, the USR similarity between the two corresponding molecules (*S*_*qt*_) is computed using eq 1, which represents the inverse scaled Manhattan distance (*d*_*M*_, eq 2) between the 12-number fingerprints of the two molecules, i.e., *K* = 12.

$$S_{qt}=\frac{1}{1+\frac{1}{K}d_{M}\left(q,t\right)}\in \left(0,1\right]$$ 1

$$d_{M}\left(q,t\right)=\overset{K}{\underset{i}{\sum }}\left|q_{i}-t_{i}\right|\in [0,\infty )$$ 2

The resulting similarity *S*_*qt*_ falls within the range (0, 1], where 0 indicates no similarity and 1 denotes identical fingerprints.

The USR method is efficient and effective for comparing molecular shapes described as cloud of indistinguishable points in 3D space. While the Cartesian coordinates inherently reflect aspects of molecular structure, USR does not consider any explicit chemical attribute, such as atomic numbers or pharmacophoric features.^49^ This design makes USR applicable to any chemical domain^55^ and very effective in specific applications.^56^ Nevertheless, for applications where specific chemical interactions are important, such as those of protein–ligand binding, augmenting shape-based representations with pharmacophoric or atom-type information boosts the identification of promising candidates in similarity-based virtual screenings.^11,57,58^ This insight has driven the development of 3D similarity methods incorporating various types of chemical information in conjunction with shape.^41,49,58–61^

Prime examples of methods going beyond shape are UFSRAT^62^ (Ultrafast Shape Recognition with Atom Types) and its successor USRCAT^58^ (USR with Credo Atom Types), which, in addition to the all-atoms USR shape fingerprint account for the shape of four subsets of atoms of specific types: hydrophobic, aromatic, hydrogen bond donor, and hydrogen bond acceptor. Consequently, their fingerprint is a collection of 60 moments: 12 moments of the complete set of atoms, and four sets of 12 moments, each based on the point cloud of a distinct atom type. These fingerprints offer a more detailed representation of molecular properties than USR fingerprints by distinguishing molecules of similar shape, but which display subtle differences in the arrangements of certain atom types. These atom types have been specifically chosen for their significance in protein–ligand interactions.^63^ For other applications of molecular similarity metrics, such as catalysis, the selected atom types may be much less relevant. More versatile methods that can enable applications in fields far beyond drug design are thus called for.

Another major limitation of USR, UFSRAT, and USRCAT is their incapability to distinguish enantiomers. This capability has been enabled in subsequent methods using vector cross products, which are invariant under reflection. For example, in the Chiral Shape Recognition (CSR)^64^ method, which builds on USR, one reference point is obtained via the cross product of the vectors *ctd–ftc* and *ctd–ftf* (Figure 1). Each enantiomer, while sharing d*_ctd_*, d*_ftc_*, and d*_ftf_* with the other enantiomer, will have one unique distance distribution involving the cross-product-generated reference point. The two differing distributions result in different fingerprints and in a less-then-unity similarity score for any pair of enantiomers.

In contrast, in the USR:OptIso method,^65^ the reference points, distance distributions, and moments of USR are kept intact. Chirality distinction is, instead, ensured by adding a 13th descriptor to the fingerprint. The extra descriptor is derived from a scalar triple product of the vectors *ctd–ctc*, *ctd–ftc*, and *ctd–ftf*, which, like the above cross product of CSR, is not invariant under reflection.

Building on CSR, the ElectroShape^59^ method has been developed to consider, in addition to the 3D coordinates, up to two additional atomic properties on each atom: partial charge and lipophilicity.^66^ The 3D molecular representation of USR and CSR is thus effectively expanded to four (4D) or five dimensions (5D), depending on whether one or two additional properties are considered. By including both 3D structural and physicochemical atomic properties, ElectroShape improves the ability to discern subtle differences in spatial distributions of charge and/or lipophilicity. The reference points are defined as for the 3D CSR method, with the coordinates of the fourth and fifth dimensions determined heuristically.

The use of heuristic procedures to define reference points is a feature common to all the existing moment-based methods. While these procedures are generally effective, they are, however, not without limitations and ambiguities. First, reference points chosen to coincide with atoms of the system lead to discontinuity: Small variations in the geometry, such as in many conformational changes, may result in a different atom being selected, resulting in a discrete and substantial change in the interatomic distances and, hence, in the moments. Minor variations between molecules can thus lead to disproportionately large differences in the resulting similarity score.^59,65^

Furthermore, methods such as CSR and ElectroShape, which use cross products to define one of the reference points, may suffer from inconsistencies. The two vectors of the cross product are defined using reference points (*ftc* and *ftf*) that may be interchangeable by symmetry. In such a scenario, the magnitude and orientation of the cross product depend on the order of the atoms in the input structures and on the algorithm used for selecting reference points. This leads to variability in the similarity scores not corresponding to true structural differences. See Section S.3 of the Supporting Information for an example and for further explanation.

Moreover, moment-based similarity methods have primarily been tailored to design of organic drug molecules.^45,58,59^ Adaption to other chemical systems is difficult. For example, USRCAT uses atom types suitable for drug design, but, to our knowledge, customization of these atom types to suit other applications would not be straightforward in any of the existing implementations. The open-source packages RDKit^67^ and Open Drug Discovery Toolkit (ODDT)^68^ do include moment-based similarity methods such as USR, USRCAT, and ElectroShape (the latter exclusively in ODDT). Customization of these tools to nonstandard applications, although certainly possible, would still require significant investments of time and effort to understand and modify the source code.

The foregoing challenges of existing methods and implementations highlight the need for a versatile moment-based method integrating the strengths of existing techniques with the flexibility to tailor the definition of similarity to specific applications. To this end, we here present the Hypershape Recognition (HSR) framework that is designed to offer the scalability and generality needed to consider any combination of atomic descriptors in 3D shape similarity comparisons. The HSR framework handles any chemical system and can be tuned to any application-specific needs, thereby broadening the scope and applicability of moment-based similarity metrics far beyond drug design.

## Methods

The Hypershape Recognition (HSR) method views chemical entities as point clouds, called *hypershapes*, in *N*-dimensional space. These N-dimensions consist of the three Cartesian coordinates and the atomic features (descriptors) that are added as independent dimension, so that *N = 3 + F*, where *F* is the number of features. Although being more flexible in terms of features, HSR shares two key characteristics with other moment-based methods. First, HSR compresses the features of each *hypershape* into a fingerprint containing an ordered list of *3­(N+*1*)* numerical values (i.e., the statistical moments of *N+*1 distributions of distances). Second, it calculates the degree of similarity using the Manhattan distance between the two fingerprints. In the following, we describe the details that make HSR unique and tunable.

The first major difference compared to other methods is that HSR can consider any atomic features expressed as a numerical value for each atom. These values can be very different from, and have other units than, the coordinates. HSR allows any conversion, tapering, or other modification of the feature value to be provided as input. Just as for the choice of the atomic features themselves, any such numerical preprocessing operation can be customized for a given application scenario (see the Atomic Features subsection) but is always applied consistently for the entire set of chemical entities being compared.

The second major difference compared to existing moment-based methods pertains to the reference points used to generate distance distributions and their moments. To address the above-mentioned ambiguities in the choice of reference points and the resulting discontinuities in the similarity metrics, HSR uses a deterministic instead of a heuristic approach for defining reference points. HSR obtains the location of the reference points by performing a Principle Component Analysis (PCA)^69^ of the centered *N*-dimensional coordinates and features. More precisely, the molecule’s principal components (PCs) are obtained via eigendecomposition of the covariance matrix of the *N*-dimensional coordinates and features. Important, the PCs are ordered in a descending order of significance, starting with the component that accounts for the greatest variance as determined by the eigenvalue with the largest absolute value. Thus, the directions defined by the PCs can be used to place reference points that are guaranteed to capture the largest possible variance in the *hypershape*.

A notable feature of PCs is that two antiparallel vectors are both solutions of the eigendecomposition.^69^ To ensure deterministic and unambiguous definition of reference points, the orientation of the PC vectors is defined using the projections of the *hypershape* onto the eigenvectors to give PCA scores. Each PC is selected to be oriented so that the largest projection onto it (its PCA score) is positive (see Subsection S.1.1 of the Supporting Information). If the largest projection belongs to symmetric points, i.e., pairs of points with projections of identical magnitude but opposite sign, the orientation of the PC must instead be determined by the second largest projection, or the third, and so forth, until a discriminant projection is found. In the scenario where no discriminating projection exists, the *hypershape* has a symmetry plane orthogonal to the PC. Such a symmetry plane is present, for example, in acetylene, in which the first PC is parallel to the molecular axis and its sign does not matter. Whether positive or negative, the distribution of distances from a reference point along this PC remains the same.

Once deterministically oriented PCs are identified, the reference points can be defined by placing one point along each PC, in addition to one at the geometrical center, i.e., the center of the PC coordinate system. Hence, the number of reference points is *N+*1 and is, therefore, dependent on the number of user-defined atomic features (descriptors). The distance with which the reference points are placed from the geometrical center of the N-dimensional *hypershape* can be adjusted. By default, each point is placed at a distance from the center equal to the largest coordinate of the atoms on that PC (see Figure 2).

**Figure 2:**
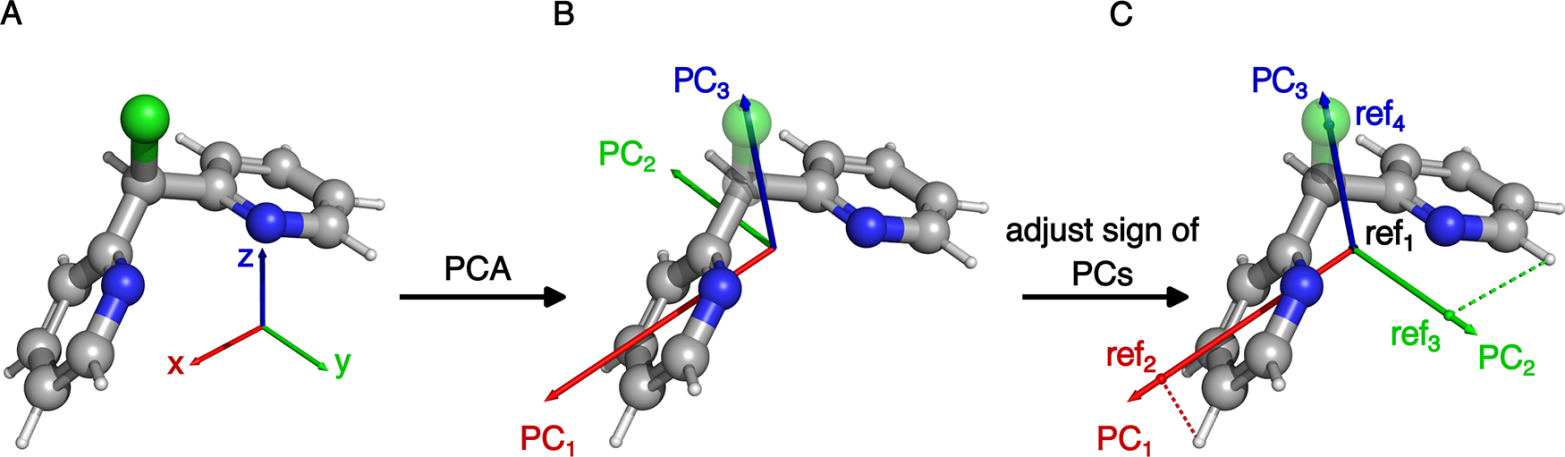
HSR reference points illustrated for a 3D representation based on the Cartesian coordinates alone. Additional atomic features (descriptors) have been omitted for clarity. (A) The starting molecule (*hypershape*) in its original Cartesian coordinate system. (B) Principal component analysis (PCA) on the centered Cartesian coordinates produces a new reference system of principal components. (C) The orientation of each PC is, if necessary, adjusted so that the largest projection onto it (its PCA score) is positive (see Subsection S.1.1 of the Supporting Information). With this deterministic orientation of the PC-based reference system established, the four reference points can be defined: the geometrical center (ref_1_), and the largest positive projection of the atomic coordinates along each principal component (ref_2_, ref_3_, and ref_4_).

Once the reference points have been defined, HSR uses those points as existing moment-based methods do. The distances between each reference point and the individual atoms of the system in N-dimensional space form the distance distributions from which the first three statistical moments are determined: the mean, the standard deviation, and the skewness. The triplets of moments are collected in an ordered fingerprint: The three moments of the geometrical center come first, followed by the corresponding triplets of each reference point according to the order of the PCs, thereby obtaining a vector of *3­(N+*1*)* numbers. For example, for a six-dimensional system involving the three Cartesian coordinates plus three additional features (descriptors) of each atom, a 21-number fingerprint is obtained. The similarity between two molecular fingerprints is determined using the inverse normalized Manhattan distance (eq 1), just as in the existing methods.

### Atomic Features

Users can include atomic features (descriptors) as additional dimensions to define the *N*-dimensional *hypershape* representation of the chemical entities. Such features can be extracted from the input representation, i.e., an RDKit’s Mol class object, or can be given directly providing the *N*-dimensional matrix representation of the *hypershape*.

In its default implementation, the HSR method incorporates 3 atomic features to produce a 21-dimensional fingerprint:

The square root of the number of protons *(p).*

$$F_{1}=\sqrt{p}$$ 3

The square root of the difference between the number of neutrons *(n)* and the number of neutrons of the most common isotope *(n*_*ci*_ *)* of the element, adjusted by the sign of the difference.

$$F_{2}=\mathrm{sign}\left(n-n_{ci}\right)\cdot \sqrt{\left|n-n_{ci}\right|}$$ 4

The formal charge (*q*) of the atom.

$$F_{3}=q$$ 5

These features, combined with the Cartesian coordinates, provide a six-dimensional representation of any arrangement of atoms. As mentioned above, a key consideration when integrating such additional features is the potential disparity in magnitude and unit compared to the 3D coordinates, a concern already noted by other developers.^59,66^ By default, HSR uses the inverse unit (1*/unit*) as the conversion constant, equating different units for simplicity. This standardization strategy, while streamlining the integration of various units, can be fully controlled by applying any customized function to the raw value of atomic descriptors. In fact, there is no perfect, first-principles-based, way in which to handle these values. For a similarity measure applied to a fixed set of chemical entities, autoscaling (i.e., mean-centering followed by scaling to unit variance) all dimensions may be a good strategy. For a generally applicable similarity measure, however, autoscaling one or more atomic features of each *hypershape* independently may lead to loss of information. The absolute atomic number is lost, for example, if the number of protons is autoscaled. In such cases, the number of protons will not help distinguish binary compounds such as PH_3_ and NH_3_. In principle, such distinction could be preserved in a pairwise autoscaling, but this would require recalculation of the fingerprints for each similarity evaluation.

In the absence of generally applicable procedures such as autoscaling, users are thus free to manipulate the values of the features to suit their own needs in terms of molecular size and features. In the default HSR implementation defined by eqs 3–5, we have attempted, by using square roots, to make the numerical values of the features associated with the number of protons and neutrons fall within practical intervals that resemble those of centered spatial coordinates for mid- and small-size molecules.

**Figure 3:**
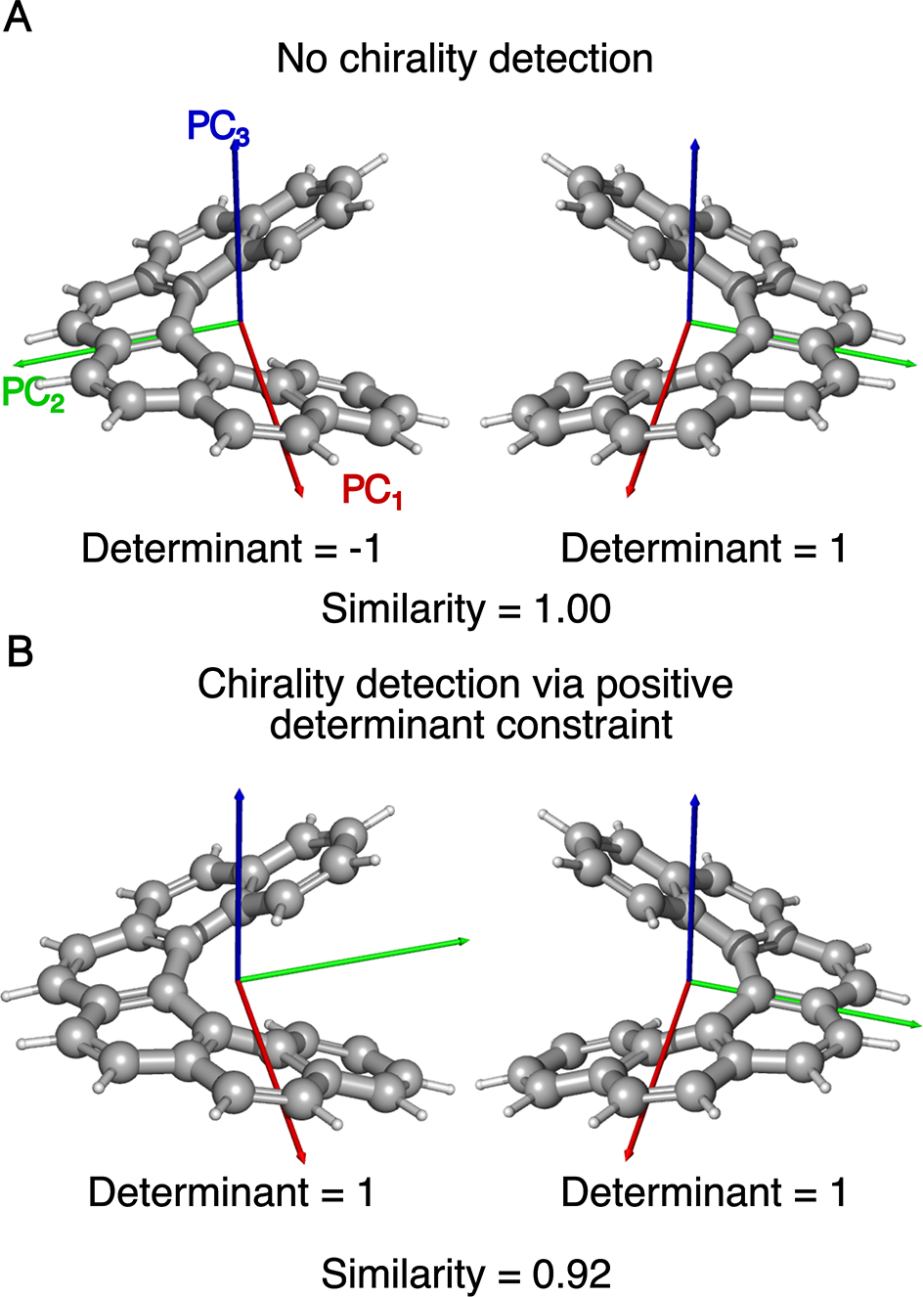
(A) The enantiomeric PCs of two hexahelicene enantiomers obtained by the chirality-insensitive HSR method. (B) PCs of the same hexahelicene enantiomers obtained by the chirality-sensitive HSR method, which flips the sign of one of the PCs of the negative-determinant enantiomer so that the determinant becomes positive.

### Chirality

With the PCs oriented as described above, two mirror-image chemical entities will have PC-based reference systems that are mirror images of each other. This leads to identical distance distributions and fingerprints. HSR can, however, optionally distinguish chiral objects. Two enantiomeric PC-based reference systems have determinants of opposite signs for the normalized eigenvectors matrix obtained from the covariance matrix. In HSR, this determinant is thus used, similarly to the reflection-invariant cross products and triple products in existing chirality-aware methods like CSR^64^ and USR:OptIso,^65^ to alter the set of reference points to obtain an enantiospecific distribution of distances. This is achieved by orienting the PCs according to (i) the sign of the determinant of the normalized eigenvectors matrix obtained from the covariance matrix (*det*_*i*_) prior to any reorientation of PCs (Figure 2B), and (ii) the number of sign changes *n*_*c*_ performed to reorient the PCs. For example, a single sign change (*n*_*c*_ = 1) suffices to produce the deterministic orientation in Figure 2C from the ambiguous orientation in Figure 2B. Notably, the determinant of the deterministic orientation without chirality distinction (*det*_*f*_), such as in Figure 2C, corresponds to

$$\det_{f}=\det_{i}\cdot {\left(-1\right)}^{n_{c}}$$ 6

If *det*_*f*_ = – 1, the sign of the eigenvector corresponding to the PC with the most skewed scores is changed to impose a determinant of +1. For a more in-depth explanation of how HSR detects chirality, see Section S1 of the Supporting Information.

Requiring a positive determinant ensures that the reference systems of two enantiomers are not mirror images anymore (see Figure 3). Calculation of the fingerprint can thus proceed as explained above for the chirality-insensitive HSR treatment. With one of the reference points differing between the two enantiomers, the resulting distances and fingerprints differ as well.

## Results and Discussion

Our aim is to establish a truly general framework for moment-based similarity methods. Hence, whereas the capability to handle inorganic and organometallic molecules and to discriminate enantiomers will be covered later in the section, we will begin by assessing HSR’s performance using the Directory of Useful Decoys-Enhanced (DUD-E). This serves to test to what extent HSR can emulate existing moment-based similarity methods within their typical application context, i.e., virtual screening of organic drug molecules. We will also consider the additional computational cost of HSR compared to the USR method and the extent to which HSR, by not explicitly using atomic positions as reference points, may overcome the above-mentioned discontinuity problems of existing methods. Finally, toward the end of the section, we will discuss the implications of introducing additional atomic features.

### DUD-E

The application of HSR in virtual screening was tested by performing benchmark tests using the Directory of Useful Decoys-Enhanced (DUD-E)^70,71^ (Table[Table tbl1]). The DUD-E enrichment factors obtained with HSR’s emulation of USR,^49^ USRCAT,^58^ and ElectroShape^59^ were compared with those from a retrospective benchmark,^58^ those obtained using the RDKit^67^ implementations of USR^49^ and USRCAT,^58^ and those obtained using the Open Drug Discovery Toolkit (ODDT)^68^ implementations of USR,^49^ USRCAT,^58^ and ElectroShape (ODDT provides the only freely available implementation of ElectroShape; it includes ODDT-computed partial charges but not lipophilicities).^59^ In these experiments, HSR emulated the functionality of each method, but employed its deterministic, PCA-based approach to define reference points instead of the heuristics of the original implementations (see Section S5.1 of the Supporting Information).

**Table 1:** DUD-E Enrichment Experiments for Selected Implementations of Similarity Methods

	enrichment factors (0.5%)^a^			
similarity method	Schreyer et al.^b^	RDKit	ODDT	HSR
USR	6.71	5.44	4.52	6.84^b^
USRCAT	11.99	10.95	9.81	11.58^c^
ElectroShape	11.27	n.a.	10.27	9.21^d^

a Ref 70.

b Ref 58.

c Obtained using HSR’s emulations of USR and USRCAT considering Cartesian coordinates only (3D *hypershape*).

d Obtained using HSR’s emulation of ElectroShape considering Cartesian coordinates and unscaled partial atomic charges (4D *hypershape*), see Section 4.4 in the Supporting Information.

In the case of USR and USRCAT, the HSR emulation delivers enrichment factors that are consistent with those of the retrospective benchmark^58^ as well as with the RDKit and ODDT tests performed here. Notably, HSR’s emulation of USR achieves the best enrichment factor among all tested implementations. In contrast, HSR offers a somewhat lower ElectroShape enrichment factor than the other implementations. This discrepancy can be attributed to the scaling between the 3D coordinates and the partial charge that is optimized in ElectroShape. This scaling is used in the ODDT implementation of ElectroShape and not in HSR. Whereas HSR allows for such scaling to be provided by the user, the emulation test performed here illustrates that even unscaled partial atomic charges used as the fourth dimension deliver an enrichment factor close to that of ElectroShape.

In summary, Table[Table tbl1] demonstrates that HSR, despite its radically different algorithm used for defining the reference points, can emulate USR, USRCAT, and ElectroShape similarity metrics. Using the principal components (PCs) to define reference points is, therefore, shown to be a reliable alternative to the heuristics of USR, USRCAT, and ElectroShape.

### Computational Complexity

While the previous paragraph demonstrated that the PCA treatment provides results comparable to the heuristic identification of the reference points, PCA introduces an additional computational cost compared to USR-based methods. In the latter, the reference points are identified by iterating through the list of atoms to select the atom with the smallest or largest distance from a given point, which has a computational complexity of *O*(*A*) (*A* being the number of atoms), i.e., its execution time increases linearly with the number of atoms. The PCA algorithm instead has a complexity of about *O*(*A* · *N* ^2^ + *N* ^3^),^72,73^ where *N* is the dimensionality of the system, i.e., the total number of coordinates and features. This means that HSR is more costly than USR and that the additional cost grows rapidly when features in addition to the Cartesian coordinates are considered. For 3D and 4D cases in which HSR emulates USR,^49^ USRCAT,^58^ and ElectroShape,^59^ the added cost of PCA appears to be small or moderate, with HSR DUD-E runtimes being comparable to those of another Python implementation, ODDT^68^ (Table S4). Further tests indicate that HSR runtimes increase moderately when extending *hypershapes* from 3D to 6D, with the latter taking about 40% longer for fingerprint calculation. Computing the fingerprint of one 6D *hypershape* takes on average 3.5 ms, and computing the similarity score given two fingerprints takes on average 14 μs (See Section S5.2 of the Supporting Information for more details on runtime experiments).

### Continuity

Despite its additional computational cost, the capability to identify reference points that do not necessarily correspond to atoms of the system makes the HSR similarity values more continuous than those of USR-based methods. The latter methods place reference points only or mostly on atoms, which makes them very sensitive to conformational changes.^59,65^ Even slight geometrical changes may introduce a new reference point, resulting in an abrupt change in the associated distance distribution and similarity score, as illustrated in Figure 4. Using our own implementation of USR,^49^ validated against the RDKit implementation (see Section S5.3 of the Supporting Information), we analyzed a previously reported discontinuity example.^65^ The USR similarity score for two molecules that differ only by a methyl substituent on the indolinone ring changes substantially from one conformer (Figure 4B) to another (Figure 4D). In contrast, the corresponding HSR scores (i.e., computed using 3D coordinates only to mimic USR) are similar and consistent with a limited, smooth change from one conformer (Figure 4C) to the next (Figure 4E).

**Figure 4:**
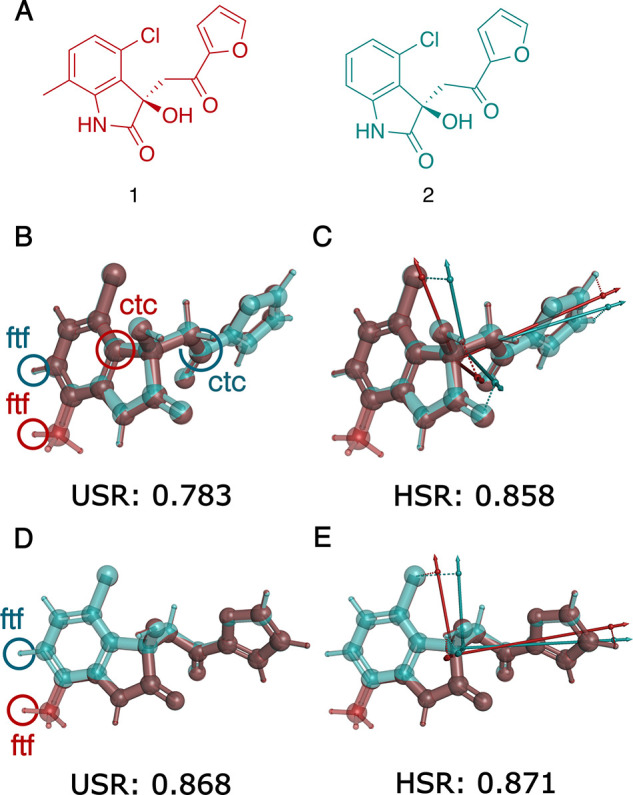
Comparison of USR and HSR analyses of two molecules that differ only by a methyl group on the indolinone ring (A) in two selected conformations, one shown in B and C and the other in D and E, respectively. The reference points and the similarity scores are calculated based on 3D spatial coordinates only using USR (B and D) and HSR (C and E). Also shown in C and E are the HSR-derived principal components for **1** (red) and **2** (turquoise), respectively. Labels of selected USR reference points: *ctc* (the atom closest to the centroid) and *ftf* (the atom farthest from *ftc*, which, in turn, is the atom farthest from the centroid. In B–E structures of **1** and **2** with the same conformation were superimposed in PyMol by aligning the common substructure.

The discontinuity observed for USR can be understood by noting that for the first conformation (Figure 4B), two reference points, *ctc* and *ftf*, are located on different atoms in the two molecules. In comparison, for the second conformation (Figure 4D), only one reference point, *ftf*, changes location between the two molecules, resulting in a substantially higher similarity score.

In contrast, HSR deals with the discontinuity problem inherent to all USR-based methods by placing reference points in positions that do not necessarily coincide with atomic positions. HSR reference points will thus move more smoothly and continuously between highly related geometries such as those of conformers.

### Inorganic Molecules

To assess the capability of HSR to handle a wide variety of molecules, we compared the similarity scores of HSR, USR, and USRCAT for pairs of inorganic and organometallic molecules.

We started by exploring the potential advantages of adding atomic features such as atom types (USRCAT), formal atomic charge (default HSR), and atomic number (default HSR) to a geometry-only approach (USR). Figure 5 presents the USR, USRCAT, and default HSR similarity comparisons of three octahedral hexaammine complexes, [Co­[NH_3_]_6_]^2+^ (Cambridge Structural Database (CSD)^74^ entry: CAFWEM), [Co­[NH_3_]_6_]^3+^ (ADIYES), and [Ir­[NH_3_]_6_]^3+^ (BIWHEY). Combined, these comparisons probe the effects of two transition metals and two oxidation states in structural analogues. Importantly, CSD^74^ structures were used to limit geometrical differences to those of the experiments and to avoid artifacts introduced by computational modeling. Such artifacts may mask geometrical responses to changes in oxidation state and other properties and were here avoided to eliminate any disadvantage for USR.

**Figure 5:**
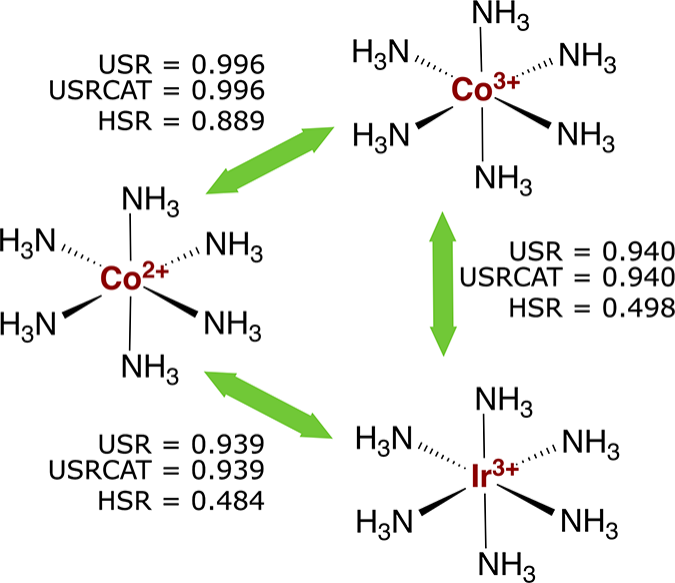
USR, USRCAT and HSR (default 6D representation) similarity comparisons of octahedral hexaammine complexes of cobalt­(II), cobalt­(III), and iridium­(III).

Indeed, USR can distinguish between [Co­[NH_3_]_6_]^2+^ and [Ir­[NH_3_]_6_]^3+^ as well as between [Co­[NH_3_]_6_]^3+^ and [Ir­[NH_3_]_6_]^3+^, as shown by a similarity score (0.94) clearly below 1 for both pairs (Figure 5). These pairs involve transition-metal ions of different rows. The importance of the central metal ion is clearly reflected in the geometries. In particular, the metal–N distances are 6%–7% longer in the iridium complex than in the cobalt complexes, which enables the geometry-based distinction in USR.

In contrast, as shown by a similarity score (0.996) only marginally lower than 1 for [Co­[NH_3_]_6_]^2+^ and [Co­[NH_3_]_6_]^3+^, oxidation state changes may be insufficiently reflected in the geometries for USR to distinguish between otherwise analogous complexes. In this case, the geometries of the two cobalt complexes are very similar, with the Co­(III)–N distances being only ca. 0.4% shorter than the Co­(II)–N ones. For USR similarity scores approaching 1, such as here, distinguishing between signal (truly different molecules) and noise (structural inaccuracies) may be difficult. With control of, and variations in, oxidation state being key to many of the unique properties of transition-metal complexes, this difficulty limits the scope of the 3D-only USR method for such compounds. Fortunately, oxidation state changes can be handled by going beyond 3D: Resolution between the two cobalt complexes is obtained by including formal atomic charges, as shown by an HSR score (0.889) communicating that the two complexes are similar but not identical.

In the above comparisons, the Credo atom types remain unchanged across the complexes, and USRCAT relies on geometrical information only. Thus, USRCAT reproduces the USR scores, and the above discussion of the performance of USR equally applies to USRCAT.

Another application for which geometrical information alone may not suffice is that of distinguishing between isomers. Figure 6A illustrates a case of linkage isomerism, where the two molecules differ only by the atom, nitrogen or oxygen, by which the nitrosyl ligand binds to the transition metal atom. Because the geometries of the two molecules are almost identical, the USR score is close to unity. The default HSR implementation, chiefly by including the element identity, differentiates between the two molecules while still highlighting their high degree of similarity. In contrast, the USRCAT score is perhaps lower than what might be expected. The lower similarity presumably originates from changes in the nitrosyl nitrogen and oxygen atom types resulting from the two ligand binding modes. USRCAT’s assignment of atom types depends on how the bonds are defined in each molecule.^20^ For inorganic and organometallic molecules that do not conform to standard valence rules, automated classification of bonds, such as in USRCAT, may lead to inconsistent comparisons. For instance, the USRCAT comparison of two copies of the same molecule differing only by formal connectivity may result in a similarity score artificially lower than 1 (see Section S5.3.3 of the Supporting Information for an example). In contrast, since HSR does not intrinsically depend on the molecular connectivity, it can be safely used for comparing molecules with ambiguous formal bond classifications.

**Figure 6:**
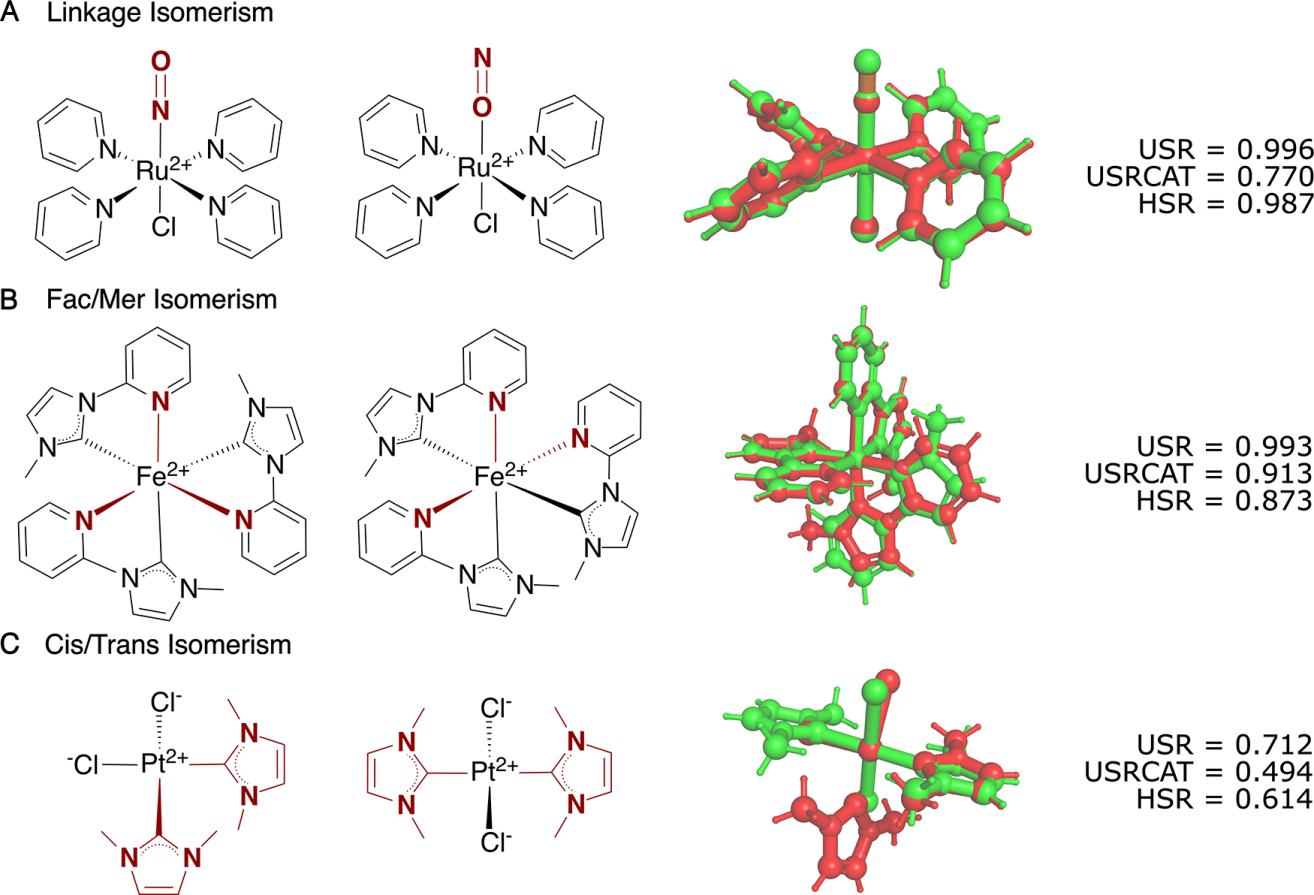
Comparison of USR,^75^ USRCAT (RDKit), and HSR (default 6D representation) similarity scores for pairs of linkage (A), *fac/mer* (B) and *cis/trans* (C) isomers. The molecules are presented both as line structures and as superimposed 3D structures. Structures obtained from the Crystallography Open database (COD),^76,77^ see Section S4.6 of the Supporting Information for details. Isomers were superimposed with PyMol considering all atoms.

In Figure 6B, the molecules differ in the stereochemical arrangement of ligands around the metal center (*fac/mer* isomerism). For USR, the lack of chemical context, again, leads to a similarity score that is too high to ensure clear distinction. In contrast, both USRCAT and HSR appear to be more sensitive to the relative arrangement of functional groups and are better able to distinguish between the isomers.

In Figure 6C, the structural change due to cis/trans isomerism is already well captured by USR. HSR, as in the previous examples, predicts a somewhat lower similarity score thanks to its sensitivity to atomic features. Similarly, USRCAT again produces a considerably lower similarity score than both HSR and USR. In this case, however, the reduction does not seem to originate from modified atom connectivity. Instead, USRCAT’s focus on atom types glorifies the stereochemical difference because, although only the four nitrogen atoms match any of the Credo atom types (i.e., hydrogen bond acceptors), the change of the relative positioning of such few atoms results in large variations of the associated moments. Such variations in the H-bond acceptors’ component account for about 60% of the modified Manhattan distance used to compute the similarity score,^58^ and lead to the drastic lowering of the score compared to USR.

### Chirality

To test whether HSR can distinguish the members of pairs of enantiomers, we computed HSR similarity scores for a collection of enantiomers assembled to cover different types of chirality (Table 2).^65^ Whereas USR and the chirality-insensitive HSR would predict the members of these pairs to be identical, the lower-than unity scores in Table 2 demonstrate that, with chirality detection turned on, HSR is indeed able to distinguish between members of such pairs.

**Table 2:**
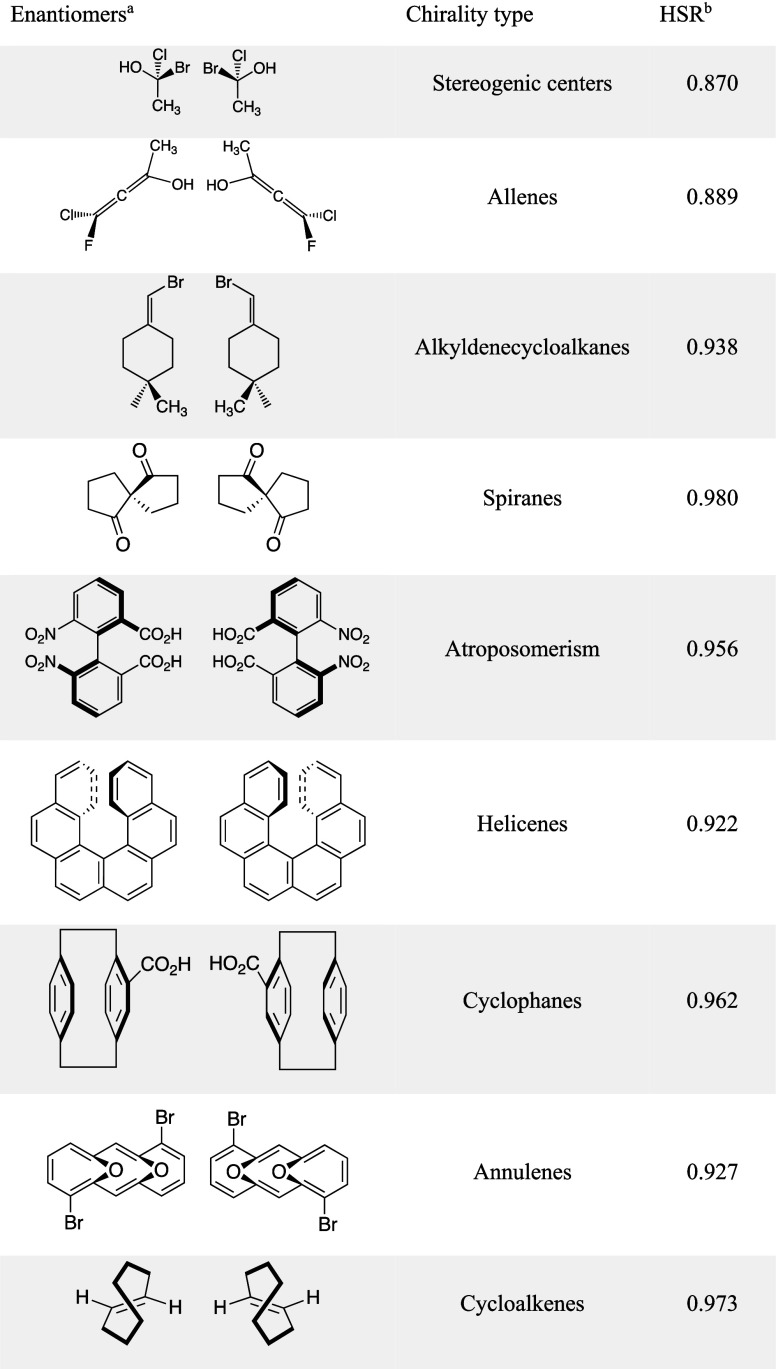
Chirality-Sensitive HSR Scores for a Variety of Enantiomeric Pairs

a See Section S4.7 of the Supporting Information for details.

b Similarity scores using only 3D coordinates of the molecules without any additional features.

Finally, consistent with our objective to extend molecular similarity analysis to inorganic and organometallic molecules, we considered the kind of axial chirality often observed in propeller-like transition metal complexes (Figure 7). CSR^75^ does distinguish between the two enantiomers, but, with a similarity score very close to unity, only barely. USR:OptIso^75^ and HSR both signal more clearly that the two enantiomers are indeed different molecules. Including atomic features magnifies the difference, as evident from the lower 6D than 3D HSR similarity score.

**Figure 7:**
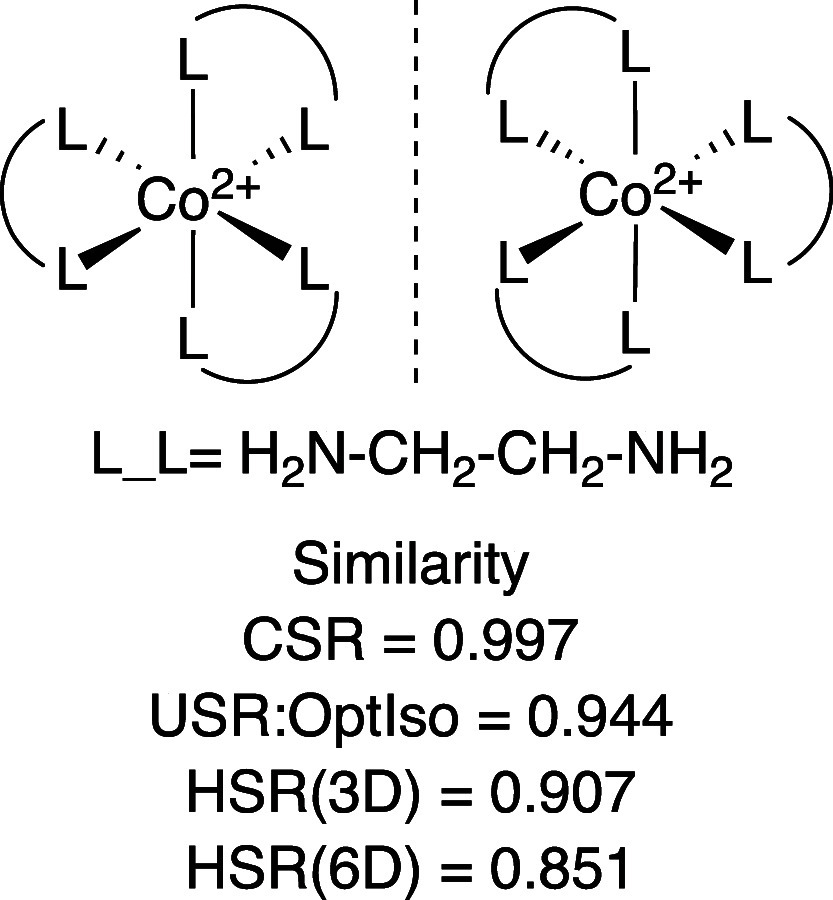
HSR (3D and 6D) similarity scores for propeller-like enantiomers (axial chirality), compared to CSR and USR:OptIso.

Although HSR can discriminate between members of enantiomeric pairs, chirality is turned off by default, for two reasons. First, chirality detection only makes sense for molecules that are described by the same number and type of dimensions, such as nonplanar structures with proton count as the sole additional feature. This requirement will, in general, not be guaranteed. In fact, *hypershapes* with varying numbers of significant PCs may easily occur, and be compared using HSR, even if the user-defined dimensionality is the same. A molecule with a constant value for all atoms along one dimension, which is the case, for example, for planar, isotopically uniform, or neutral molecules, will have one less significant PC than an otherwise identical molecule that has a slight variation in value along the same dimension. Since such molecules are not enantiomers and it does not make sense to invoke a determinant-based orientation of their reference system, chirality detection is turned off by default.

A second reason for invoking chirality detection only when strictly necessary pertains to comparison of objects across different kinds of optical isomerism. While HSR can discriminate between members of enantiomeric pairs, as illustrated in Table 2, it does not identify the specific type of chirality. Thus, for molecules of different kinds of optical isomerism, chirality-sensitive similarity scores are likely to be inconsistent and difficult to interpret.

Concluding, while HSR’s ability to distinguish between enantiomers is a potent feature of the method, users should be aware that including chirality information in similarity assessments may be beneficial only in specific contexts.

### Effect of Atomic Features

The capability of the HSR framework to include any additional atomic features, i.e., to extend the dimensionality of the *hypershape*, has been designed to enhance the discriminatory power of the similarity assessment. Whereas any added feature should, intuitively, increase this power, the response, in terms of similarity score, to feature additions may sometimes be more complex, as we will discuss in the following.

First, if a discriminating feature is independent from all the other coordinates and features, its effect is indeed the expected increase in discriminatory power. For example, whereas a 3D- (only spatial coordinates) or 4D-representation (spatial coordinates and atomic number, via eq 3) cannot distinguish a ^13^C-labeled molecule from its ^12^C-only isotopologue (Figure 8A), the necessary discriminatory power is achieved by adding isotope information (eq 4) as the fifth dimension, as evident from both the Manhattan distance between the fingerprints and the HSR similarity score (Figure 8C). The effect of the labeling on the Manhattan distance and the similarity score decreases with the size of molecule, however, as the labeled site makes up an ever-smaller fraction of the molecule.

**Figure 8:**
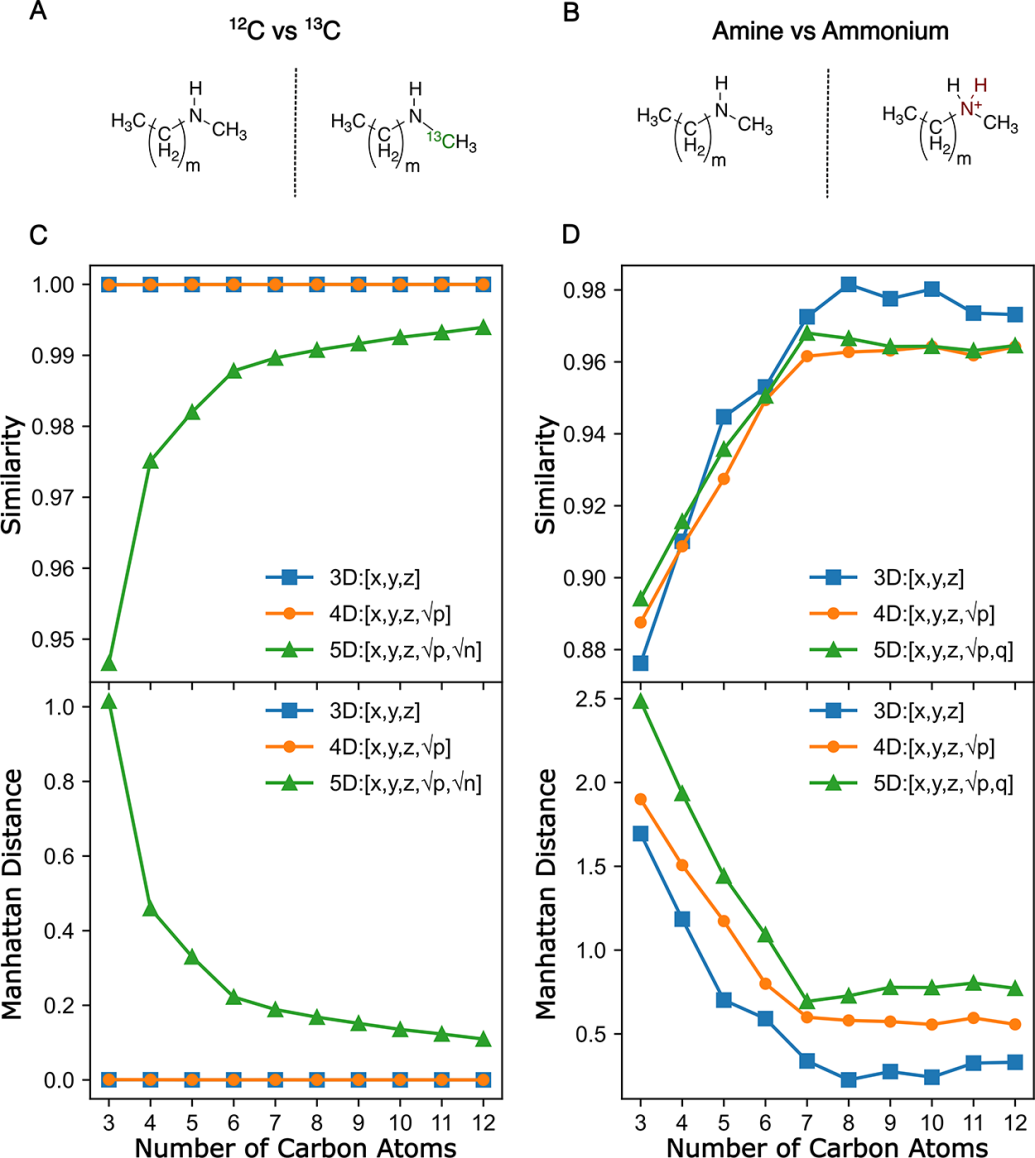
^13^C labeling (A) and protonation (B) of amines of increasing size and the corresponding effects on the HSR similarity score (C and D, respectively). The 3D representation only includes the spatial coordinates, whereas the 4D and 5D representations also include the atomic number (eq 3) and the atomic number as well as isotope information (eq 4), respectively.

Second, the response to a feature addition may be more complex if multiple features describe the difference between the two molecules. For example, protonation of an amine alters the formal charge of the nitrogen atom (Figure 8B), which may be explicitly recorded as a feature (eq 5). At the same time, protonation introduces a new atom, which will be reflected in changes to both the spatial coordinates and the element feature (eq 3). Protonation is, therefore, a complex modification of the *hypershape*, affecting up to 5 of the 6 dimensions. The modification is therefore captured by the 3D, 4D, and 5D (spatial coordinates, atomic number, and formal charge, via eq 5) representations alike (Figure 8B and Figure 8D). The similarity scores, however, do not follow the intuitive 3D > 4D > 5D order, and the differences between the three series do not follow a clear trend that depends on the size of the molecule. To start understanding this complexity, we first note that the Manhattan distances do, in fact, follow the order 3D < 4D < 5D (Figure 8D). The trend is thus destroyed by the transformation from Manhattan distances to similarity scores (eq 1). This transformation includes a normalization constant (*K* in eq 1) that depends on the dimensionality of the molecular representation, as *K* = *3­(N+*1*),* where *N* is the dimensionality. Similarity scores produced by representations of different dimensionality are thus not directly comparable.

As a corollary, we note that the concept of distance (dissimilarity) and similarity are inextricably connected, and both are used for comparing molecules.^15^ For applications involving variations in dimensionality, using the Manhattan distances instead of the normalized similarity scores may be preferable.

Finally, it is worth noting that adding atomic features to the 3D representations disrupts the original geometrical interpretation of the first three statistical moments.^49^ When considering only the Cartesian coordinates of the cloud of points, the mean of the distance distribution (μ_1_^*ctd*^, Figure 1) informs on the size of the molecule, the variance (μ_2_^*ctd*^, Figure 1) on the compactness, and the skewness (μ_3_^*ctd*^, Figure 1) on the degree of asymmetry. When adding atomic features, this geometry-only interpretation of the HSR principal components is no longer valid.

### Position Dependency

Finally, a perhaps unexpected property of moment-based similarity methods that go beyond spatial coordinates is that the impact of a given feature depends on its position in the molecule. This can be seen, for instance, when substituting a single deuterium (^2^H) for a regular hydrogen atom (^1^H) in a molecule encompassing only regular hydrogen atoms (Figure 9). The similarity score calculated for the regular molecule and its isotopologue varies depending on which hydrogen atom that is substituted.

**Figure 9:**
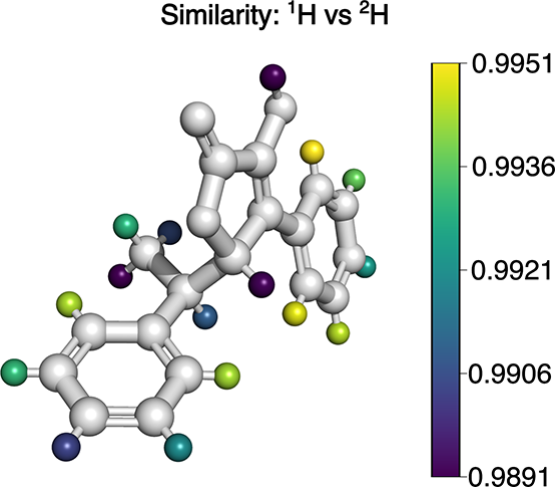
Color coding indicating the similarity between a molecule encompassing only regular hydrogen atoms and its isotopologue in which a single deuterium (^2^H) atom has substituted a regular (^1^H) hydrogen atom. The similarity scores are those of the default 6D HSR implementation.

While this position dependence is likely to be surprising to some, it is the result of a consistent and objective treatment of the molecules in terms of atomic coordinates and features. Optimizing instead for maximal alignment with the expectations of chemists could be achieved, for example, via training of machine-learning models.^78^ This approach would, in contrast to machine-learning models trained using descriptors of moment-based methods,^79^ result in models biased toward human perception and is orthogonal to the strategy followed here. Rather than catering to human intuition, HSR offers an objective and versatile framework that can be used to develop moment-based similarity methods adapted to an unmatched range of applications.

## Conclusions

The Hypershape Recognition (HSR) framework avoids the similarity score discontinuities observed for moment-based methods relying on atoms as reference points from which to calculate distance distributions and their moments. HSR, instead, places reference points on the principal components (PCs) derived from principal component analysis (PCA) of the *N*-dimensional representation, the *hypershape*, of the chemical entity (molecule) being compared. Furthermore, by requiring the determinant of the PC-based reference systems of two molecules to have the same sign, HSR may, optionally, distinguish between enantiomers.

A hallmark of HSR is that the *hypershapes* can be customized and expanded. In a general-purpose implementation, HSR includes a default similarity score that considers, in addition to the Cartesian coordinates of each atom, features, i.e., atomic descriptors, containing information about the atomic number, the isotope (the number of neutrons), and the formal charge of those atoms. This similarity score was here demonstrated to distinguish between isomers of inorganic and organometallic complexes and, as the first moment-based similarity method, also between isotopologues and redox pairs. Importantly, since the underlying HSR framework handles any numerical feature as additional dimension of the *hypershape*, HSR can be tailored for any application by providing the values of the relevant atomic features or even by defining property-annotated dummy atoms (e.g., pharmacophores). This degree of customizability is unique among 3D-similarity methods; HSR may even emulate other moment-based similarity methods, as shown here in enrichment experiments using the DUD-E set.

Overall, the HSR framework empowers users to customize moment-based similarity measures to meet their needs. HSR may thus help expand the use of similarity measures to new chemical systems and application areas. Equally important, HSR also is a foundation on which further developments and refinements of moment-based similarity measures may be built.

## Supplementary Material



## Data Availability

The Hypershape Recognition (HSR) source code is available on Github at: https://github.com/denoptim-project/HSR. The data and scripts to reproduce results and figures shown in the paper are available on Zenodo at: https://doi.org/10.5281/zenodo.14631654

## Notes

Detailed discussion of the methods for PCA and orientation of PCs, numerical examples of fingerprints and similarity score calculations, discussion of symmetry-related challenges for chiral detection methods, computational details and data for reproducing molecular modeling and similarity evaluations (PDF)

## References

[1] Maggiora G., Vogt M., Stumpfe D., Bajorath J. (2014). Molecular Similarity
in Medicinal Chemistry. J. Med. Chem..

[2] Bender A., Glen R. C. (2004). Molecular Similarity:
A Key Technique in Molecular
Informatics. Org. Biomol. Chem..

[3] Martin Y. C., Kofron J. L., Traphagen L. M. (2002). Do Structurally
Similar Molecules
Have Similar Biological Activity?. J. Med. Chem..

[4] Johnson, M. A. ; Maggiora, G. M. Concepts and Applications of Molecular Similarity; John Wiley & Sons: New York, 1990.

[5] Huang L., Luo H., Li S., Wu F.-X., Wang J. (2020). Drug–Drug Similarity
Measure and Its Applications. Briefings Bioinf..

[6] Kumar A., Zhang K. Y. J. (2018). Advances in the
Development of Shape Similarity Methods
and Their Application in Drug Discovery. Front.
Chem..

[7] Koutsoukas A., Simms B., Kirchmair J., Bond P. J., Whitmore A. V., Zimmer S., Young M. P., Jenkins J. L., Glick M., Glen R. C., Bender A. (2011). From in Silico
Target Prediction
to Multi-Target Drug Design: Current Databases, Methods and Applications. J. Proteomics.

[8] Barupal D. K., Fan S., Fiehn O. (2018). Integrating Bioinformatics
Approaches for a Comprehensive
Interpretation of Metabolomics Datasets. Curr.
Opin. Biotechnol..

[9] De S., Bartók A. P., Csányi G., Ceriotti M. (2016). Comparing Molecules
and Solids across Structural and Alchemical Space. Phys. Chem. Chem. Phys..

[10] Mubashir T., Hussain Tahir M., Altaf Y., Ahmad F., Arshad M., Hakamy A., Sulaman M. (2023). Statistical Analysis and Visualization
of Data of Non-Fullerene Small Molecule Acceptors from Harvard Organic
Photovoltaic Database. Structural Similarity Analysis with Famous
Non-Fullerene Small Molecule Acceptors to Search New Building Blocks. J. Photochem. Photobiol. Chem..

[11] Hawkins P. C. D., Skillman A. G., Nicholls A. (2007). Comparison
of Shape-Matching and
Docking as Virtual Screening Tools. J. Med.
Chem..

[12] Muegge I., Mukherjee P. (2016). An Overview
of Molecular Fingerprint Similarity Search
in Virtual Screening. Expert Opin. Drug Discovery.

[13] Cereto-Massagué A., Ojeda M. J., Valls C., Mulero M., Garcia-Vallvé S., Pujadas G. (2015). Molecular Fingerprint Similarity Search in Virtual
Screening. Methods.

[14] Maggiora G. M., Shanmugasundaram V. (2011). Molecular
Similarity Measures. Methods Mol. Biol. Clifton
NJ..

[15] Bero S. A., Muda A. K., Choo Y. H., Muda N. A., Pratama S. F. (2017). Similarity
Measure for Molecular Structure: A Brief Review. J. Phys. Conf. Ser..

[16] Quirós M., Gražulis S., Girdzijauskaitė S., Merkys A., Vaitkus A. (2018). Using SMILES
Strings for the Description of Chemical
Connectivity in the Crystallography Open Database. J. Cheminformatics.

[17] Willett P. (2006). Similarity-Based
Virtual Screening Using 2D Fingerprints. Drug
Discovery Today.

[18] McGaughey G. B., Sheridan R. P., Bayly C. I., Culberson J. C., Kreatsoulas C., Lindsley S., Maiorov V., Truchon J.-F., Cornell W. D. (2007). Comparison of Topological, Shape, and Docking Methods
in Virtual Screening. J. Chem. Inf. Model..

[19] Sheridan R. P., Kearsley S. K. (2002). Why Do We Need so
Many Chemical Similarity Search Methods?. Drug
Discovery Today.

[20] Clark A. M. (2011). Accurate
Specification of Molecular Structures: The Case for Zero-Order Bonds
and Explicit Hydrogen Counting. J. Chem. Inf.
Model..

[21] Medina-Franco J. L., Cruz-Lemus Y., Percastre-Cruz Y. (2020). Chemoinformatic Resources for Organometallic
Drug Discovery. Comput. Mol. Biosci..

[22] Mjos K. D., Orvig C. (2014). Metallodrugs in Medicinal
Inorganic Chemistry. Chem. Rev..

[23] Medina-Franco J. L., López-López E., Andrade E., Ruiz-Azuara L., Frei A., Guan D., Zuegg J., Blaskovich M. A. T. (2022). Bridging
Informatics and Medicinal Inorganic Chemistry: Toward a Database of
Metallodrugs and Metallodrug Candidates. Drug
Discovery Today.

[24] Markwalter C. F., Kantor A. G., Moore C. P., Richardson K. A., Wright D. W. (2019). Inorganic Complexes and Metal-Based Nanomaterials for
Infectious Disease Diagnostics. Chem. Rev..

[25] Meggers E. (2009). Targeting
Proteins with Metal Complexes. Chem. Commun..

[26] Takahashi K., Ohyama J., Nishimura S., Fujima J., Takahashi L., Uno T., Taniike T. (2023). Catalysts
Informatics: Paradigm Shift towards Data-Driven
Catalyst Design. Chem. Commun..

[27] Ramakrishna S., Zhang T.-Y., Lu W.-C., Qian Q., Low J. S. C., Yune J. H. R., Tan D. Z. L., Bressan S., Sanvito S., Kalidindi S. R. (2019). Materials
Informatics. J. Intell.
Manuf..

[28] Foscato M., Jensen V. R. (2020). Automated in Silico Design of Homogeneous Catalysts. ACS Catal..

[29] Kalikadien A. V., Mirza A., Hossaini A. N., Sreenithya A., Pidko E. A. (2024). Paving the Road towards Automated Homogeneous Catalyst
Design. ChemPlusChem..

[30] Ioannidis E. I., Gani T. Z. H., Kulik H. J. (2016). molSimplify:
A Toolkit for Automating
Discovery in Inorganic Chemistry. J. Comput.
Chem..

[31] Nandy A., Duan C., Kulik H. J. (2022). Audacity
of Huge: Overcoming Challenges
of Data Scarcity and Data Quality for Machine Learning in Computational
Materials Discovery. Curr. Opin. Chem. Eng..

[32] Miklitz M., Turcani L., Greenaway R. L., Jelfs K. E. (2020). Computational Discovery
of Molecular C60 Encapsulants with an Evolutionary Algorithm. Commun. Chem..

[33] Bicerano J., Rigby D., Freeman C., LeBlanc B., Aubry J. (2024). Polymer Expert
– A Software Tool for de Novo Polymer Design. Comput. Mater. Sci..

[34] Brammer J. C., Blanke G., Kellner C., Hoffmann A., Herres-Pawlis S., Schatzschneider U. (2022). TUCAN: A Molecular
Identifier and Descriptor Applicable
to the Whole Periodic Table from Hydrogen to Oganesson. J. Cheminformatics.

[35] Krenn M., Ai Q., Barthel S., Carson N., Frei A., Frey N. C., Friederich P., Gaudin T., Gayle A. A., Jablonka K. M., Lameiro R. F., Lemm D., Lo A., Moosavi S. M., Nápoles-Duarte J. M., Nigam A., Pollice R., Rajan K., Schatzschneider U., Schwaller P., Skreta M., Smit B., Strieth-Kalthoff F., Sun C., Tom G., von Rudorff G. F., Wang A., White A. D., Young A., Yu R., Aspuru-Guzik A. (2022). SELFIES and
the Future of Molecular String Representations. Patterns.

[36] Wigh D. S., Goodman J. M., Lapkin A. A. (2022). A Review
of Molecular Representation
in the Age of Machine Learning. WIREs Comput.
Mol. Sci..

[37] Willett P. (2014). The Calculation
of Molecular Structural Similarity: Principles and Practice. Mol. Inform..

[38] Willett P. (2009). Similarity
Methods in Chemoinformatics. Annu. Rev. Inf.
Sci. Technol..

[39] Bartók A. P., Kondor R., Csányi G. (2013). On Representing
Chemical Environments. Phys. Rev. B.

[40] De, S. ; Bartók, A. P. ; Csányi, G. ; Ceriotti, M. Comparing Molecules and Solids across Structural and Alchemical Space. arXiv 2020. 10.48550/arXiv.1601.04077. 27101873

[41] Grant J. A., Gallardo M. A., Pickup B. T. (1996). A Fast
Method of Molecular Shape
Comparison: A Simple Application of a Gaussian Description of Molecular
Shape. J. Comput. Chem..

[42] Tuccinardi T., Ortore G., Santos M. A., Marques S. M., Nuti E., Rossello A., Martinelli A. (2009). Multitemplate
Alignment Method for
the Development of a Reliable 3D-QSAR Model for the Analysis of MMP3
Inhibitors. J. Chem. Inf. Model..

[43] Tresadern G., Bemporad D., Howe T. (2009). A Comparison
of Ligand Based Virtual
Screening Methods and Application to Corticotropin Releasing Factor
1 Receptor. J. Mol. Graph. Model..

[44] Hoeger B., Diether M., Ballester P. J., Köhn M. (2014). Biochemical
Evaluation of Virtual Screening Methods Reveals a Cell-Active Inhibitor
of the Cancer-Promoting Phosphatases of Regenerating Liver. Eur. J. Med. Chem..

[45] Ballester P. J. (2011). Ultrafast
Shape Recognition: Method and Applications. Future Med. Chem..

[46] Ballester P. J., Westwood I., Laurieri N., Sim E., Richards W. G. (2010). Prospective
Virtual Screening with Ultrafast Shape Recognition: The Identification
of Novel Inhibitors of Arylamine N-Acetyltransferases. J. R. Soc. Interface.

[47] Ballester P. J., Finn P. W., Richards W. G. (2009). Ultrafast Shape Recognition: Evaluating
a New Ligand-Based Virtual Screening Technology. J. Mol. Graph. Model..

[48] Ballester P. J., Richards W. G. (2007). Ultrafast Shape Recognition for Similarity
Search in
Molecular Databases. Proc. R. Soc. Math. Phys.
Eng. Sci..

[49] Ballester P. J., Richards W. G. (2007). Ultrafast Shape Recognition to Search Compound Databases
for Similar Molecular Shapes. J. Comput. Chem..

[50] Ballester, P. J. Shape Recognition Methods and Systems for Searching Molecular Databases. US8244483B2, August 14, 2012.

[51] Patil S. P., Ballester P. J., Kerezsi C. R. (2014). Prospective Virtual Screening for
Novel P53–MDM2 Inhibitors Using Ultrafast Shape Recognition. J. Comput. Aided Mol. Des..

[52] Teo C. Y., Rahman M. B. A., Chor A. L. T., Salleh A. B., Ballester P. J., Tejo B. A. (2013). Ligand-Based Virtual Screening for
the Discovery of
Inhibitors for Protein Arginine Deiminase Type 4 (PAD4). Metabolomics.

[53] Polyakov V. R., Alexandrov V., Maderna A., Bajjuri K., Li X., Zhou S. (2022). Indexing Ultrafast
Shape-Based Descriptors in MongoDB to Identify
TLR4 Pathway Agonists. J. Chem. Inf. Model..

[54] Jang W. D., Jeon S., Kim S., Lee S. Y. (2021). Drugs Repurposed
for COVID-19 by Virtual Screening of 6,218 Drugs and Cell-Based Assay. Proc. Natl. Acad. Sci. U. S. A..

[55] Hsu P.-J. (2014). A New Perspective
of Shape Recognition to Discover the Phase Transition of Finite-Size
Clusters. J. Comput. Chem..

[56] Kortagere S., Krasowski M. D., Ekins S. (2009). The Importance of Discerning Shape
in Molecular Pharmacology. Trends Pharmacol.
Sci..

[57] Kirchmair J., Distinto S., Markt P., Schuster D., Spitzer G. M., Liedl K. R., Wolber G. (2009). How to Optimize Shape-Based Virtual
Screening: Choosing the Right Query and Including Chemical Information. J. Chem. Inf. Model..

[58] Schreyer A. M., Blundell T. (2012). USRCAT: Real-Time Ultrafast Shape Recognition with
Pharmacophoric Constraints. J. Cheminformatics.

[59] Armstrong M. S., Morris G. M., Finn P. W., Sharma R., Moretti L., Cooper R. I., Richards W. G. (2010). ElectroShape:
Fast Molecular Similarity
Calculations Incorporating Shape, Chirality and Electrostatics. J. Comput. Aided Mol. Des..

[60] Rush T. S., Grant J. A., Mosyak L., Nicholls A. (2005). A Shape-Based 3-D Scaffold
Hopping Method and Its Application to a Bacterial Protein–Protein
Interaction. J. Med. Chem..

[61] Kearnes S., Pande V. (2016). ROCS-Derived Features
for Virtual Screening. J. Comput. Aided Mol.
Des..

[62] Shave S., Blackburn E. A., Adie J., Houston D. R., Auer M., Webster S. P., Taylor P., Walkinshaw M. D. (2015). UFSRAT:
Ultra-Fast Shape Recognition with Atom Types – The Discovery
of Novel Bioactive Small Molecular Scaffolds for FKBP12 and 11βHSD1. PLoS One.

[63] Ferreira
de Freitas R., Schapira M. (2017). A Systematic Analysis of Atomic Protein–Ligand
Interactions in the PDB. MedChemComm.

[64] Armstrong M. S., Morris G. M., Finn P. W., Sharma R., Richards W. G. (2009). Molecular
Similarity Including Chirality. J. Mol. Graph.
Model..

[65] Zhou T., Lafleur K., Caflisch A. (2010). Complementing Ultrafast Shape Recognition
with an Optical Isomerism Descriptor. J. Mol.
Graph. Model..

[66] Armstrong M. S., Finn P. W., Morris G. M., Richards W. G. (2011). Improving the Accuracy
of Ultrafast Ligand-Based Screening: Incorporating Lipophilicity into
ElectroShape as an Extra Dimension. J. Comput.
Aided Mol. Des..

[67] RDKit: Open-source cheminformatics, 2023_09_3 (Q3 2023) Release, 2023. 10.5281/zenodo.10275225.

[68] Wójcikowski M., Zielenkiewicz P., Siedlecki P. (2015). Open Drug Discovery Toolkit (ODDT):
A New Open-Source Player in the Drug Discovery Field. J. Cheminformatics.

[69] Jolliffe I. T., Cadima J. (2016). Principal Component Analysis: A Review and Recent Developments. Philos. Transact. A Math. Phys. Eng. Sci..

[70] Huang N., Shoichet B. K., Irwin J. J. (2006). Benchmarking
Sets for Molecular Docking. J. Med. Chem..

[71] Mysinger M. M., Carchia M., Irwin J. J., Shoichet B. K. (2012). Directory of Useful
Decoys, Enhanced (DUD-E): Better Ligands and Decoys for Better Benchmarking. J. Med. Chem..

[72] Elgamal, T. ; Hefeeda, M. Analysis of PCA Algorithms in Distributed Environments. arXiv 2015. 10.48550/arXiv.1503.05214.

[73] Anowar F., Sadaoui S., Selim B. (2021). Conceptual and Empirical
Comparison
of Dimensionality Reduction Algorithms (PCA, KPCA, LDA, MDS, SVD,
LLE, ISOMAP, LE, ICA, t-SNE). Comput. Sci. Rev..

[74] Groom C. R., Bruno I. J., Lightfoot M. P., Ward S. C. (2016). The Cambridge Structural
Database. Acta Crystallogr. Sect. B Struct.
Sci. Cryst. Eng. Mater..

[75] The in-house implementations of the USR, CSR, and USR: OptIso methods are available at 10.5281/zenodo.14631654.

[76] (IUCr) Crystallography Open Database – an open-access collection of crystal structures https://journals.iucr.org/j/issues/2009/04/00/kk5039/index.html. 10.1107/S0021889809016690 PMC3253730 22477773

[77] Gražulis S., Daškevič A., Merkys A., Chateigner D., Lutterotti L., Quirós M., Serebryanaya N. R., Moeck P., Downs R. T., Le Bail A. (2012). Crystallography Open
Database (COD): An Open-Access Collection of Crystal Structures and
Platform for World-Wide Collaboration. Nucleic
Acids Res..

[78] Gandini E., Marcou G., Bonachera F., Varnek A., Pieraccini S., Sironi M. (2022). Molecular Similarity
Perception Based on Machine-Learning
Models. Int. J. Mol. Sci..

[79] Bonanno E., Ebejer J.-P. (2020). Applying Machine Learning to Ultrafast Shape Recognition
in Ligand-Based Virtual Screening. Front. Pharmacol..

