# Supplementary material for: Hypershape Recognition: A General Framework for Moment-Based Molecular Similarity
Source: J Chem Inf Model. 2025 Jun 11;65(12):5960–72. doi: 10.1021/acs.jcim.5c00555 (PMC12199306; doi:10.1021/acs.jcim.5c00555)
Supplement: Supplementary file 1 [file ci5c00555_si_001.pdf]

Supporting Information for

Hypershape Recognition: A General Framework for Moment-Based Molecular Similarity

Marcello Costamagna,[†] Marco Foscato,[†] David Grellscheid,[‡] Vidar R. Jensen[*†]*

†Department of Chemistry, University of Bergen, Bergen, Norway
‡Department of Informatics, University of Bergen, Bergen, Norway

Table of Contents

S.1. ORIENTATION OF PRINCIPAL COMPONENTS S2
S.1.1. ORIENTATION OF PCS WITHOUT CHIRALITY DISCRIMINATION S2
S.1.1.1. Demonstration S3
S.1.2. CHIRAL DISCRIMINATION ALGORITHM S5
S.1.2.1. Demonstration S7
S.1.2.1.1 Identical Molecules S7
S.1.2.1.2 Enantiomers S9
S.2. NUMERICAL EXAMPLE S12
S.2.1. DETERMINATION OF THE FINGERPRINT FOR MOLECULE *A* S12
S.2.2. DETERMINATION OF THE FINGERPRINT FOR MOLECULE *B* S16
S.2.3. SIMILARITY S20
S.3. CHIRALITY DETECTION METHODS AND SYMMETRY S21
S.4. COMPUTATIONAL DETAILS S23
S.4.1. USR OUTLINE S23
S.4.2. HSR REFERENCE POINTS S23
S.4.3. HSR CHIRALITY METHOD S23
S.4.4. DUD-E EXPERIMENTS S23
S.4.5. CONTINUITY S24
S.4.6. INORGANICS S24
S.4.7. CHIRALITY S24
S.4.8. FEATURES S24
S.4.9. POSITION DEPENDENCY S25
S.5. COMPUTATIONAL DATA S25
S.5.1. DUD-E S25
S.5.2. RUNTIMES S28
S.5.3. VALIDATION OF IN-HOUSE USR IMPLEMENTATION S28
S.5.3.1. Continuity S29
S.5.3.2. Inorganics S29
S.5.3.3. USRCAT's Connectivity Dependency S29
S.6. REFERENCES S30

S.1. Orientation of Principal Components

Principal Component Analysis (PCA) is a popular dimensionality reduction technique that, given a set of *N*-dimensional data points (i.e., each point is defined by *N* variables or coordinates), can identify Principal Components (PCs), i.e., the orthogonal linear combinations of the original dimensions that capture the largest possible variance in the dataset. This can be achieved via eigendecomposition of the covariance matrix to obtain eigenvectors (the PCs) and eigenvalues, which represent the variance captured by the corresponding eigenvectors. However, for a generic matrix $\boldsymbol{X}$, if $\boldsymbol{v}$ is an eigenvector of $\boldsymbol{X}$ with eigenvalue λ such that:

$$\boldsymbol{X}\boldsymbol{v} = \lambda\boldsymbol{v} \tag{S1}$$

then, the negation of that eigenvector $(-\boldsymbol{v})$ is also an eigenvector:

$$\boldsymbol{X}(-\boldsymbol{v}) = -\lambda\boldsymbol{v} = \lambda(-\boldsymbol{v}) \tag{S2}$$

Therefore, the signs of the eigenvectors and, hence, the signs of the PCs resulting from PCA are usually arbitrary. In fact, since each eigenvector can point in either of two directions, there are 2^N sets of differently signed PCs that can equally describe the same *N*-dimensional dataset.

Since our similarity method defines one reference point along each PC, and since such reference points must be defined consistently, the arbitrariness resulting from the sign of the PCs must be removed. The following sections explain how this is achieved, with or without the capability to distinguish between chiral sets of points.

S.1.1. Orientation of PCs Without Chirality Discrimination

To remove the arbitrariness in the orientation of the eigenvectors, we have developed a deterministic algorithm that only uses the dataset itself. The algorithm proceeds via two main steps:

1. for each PC, the projections of all points of the *hypershape* onto it, i.e., the PCA scores, are computed, (line 4 in Algorithm S1)
2. the direction of the PC is then chosen to ensure that the maximum projection value (score) not counterbalanced by a corresponding, oppositely signed, score of value equal to within 1×10^{-10} is positive (lines 7–10 in Algorithm S1).

Algorithm S1. Deterministic Orientation of Principal Components Based on PCA Scores.

1- ***Input:*** *N-dimensional matrix of coordinates* $\boldsymbol{A}$

2- *Covariance matrix of* $\boldsymbol{A}$: $\boldsymbol{cov}(\boldsymbol{A}) = \frac{1}{N}(\boldsymbol{A} - \overline{\boldsymbol{A}})^{\boldsymbol{T}}(\boldsymbol{A} - \overline{\boldsymbol{A}})$

3- *Principal Components* $\boldsymbol{V}$: *Eigendecomposition(*$\boldsymbol{cov}(\boldsymbol{A})$*)*

4- *Projection matrix/PCA scores* $\boldsymbol{P}$: $\boldsymbol{P} = \boldsymbol{AV}$

5- *For each column j in* $\boldsymbol{P}$*:*

6- | *Find index of maximum PCA score in column j:* $k = argmax(\boldsymbol{p}_{[:,j]})$

7- | | *If* $p_{k,j} < 0$*:*

8- *Flip the orientation of the j-th eigenvector in* $\boldsymbol{V}$: $\boldsymbol{v}^{\dagger}_{[:,j]} = -\boldsymbol{v}_{[:,j]}$
9- *else* $p_{k,j} > 0$:
10- $\boldsymbol{v}^{\dagger}_{[:,j]} = \boldsymbol{v}_{[:,j]}$
11- ***Output:*** $\boldsymbol{V}^{\dagger}$

The process of flipping the orientation of the eigenvectors, steps 7–10 in Algorithm S1, can be expressed mathematically as the application of a reflection matrix ($\mathbf{Q}$) to the principal components $\boldsymbol{V}$ to yield $\boldsymbol{V}^{\dagger}$, i.e., $\boldsymbol{V}^{\dagger} = \mathbf{VQ}$.

The matrix of signed PCs ($\boldsymbol{V}^{\dagger}$) resulting from application of the above algorithm establishes a reproducible and unambiguous reference system for the dataset. This matrix effectively represents a transformation that reliably maps any given dataset to a consistent set of coordinates in an unambiguous reference system of PCs, irrespective of the dataset's initial orientation. Thus, even when the same collection of *N*-dimensional points is presented in different orientations or order, the corresponding transformation matrix $\boldsymbol{V}^{\dagger}$ always yields an identical set of transformed coordinates for each point.

S.1.1.1. Demonstration

Consider two identical molecules *A* and *B* with *M* atoms, the *N* coordinates of which are collected in two *M*×*N* matrices, $\boldsymbol{A}$ and $\boldsymbol{B}$. The coordinates comprise the 3D Cartesian coordinates plus any number of atomic features (descriptors). For the sake of simplicity, but without any lack of generality, we assume that two molecules have identical geometry, and that the geometric center of each molecule coincides with the origin of the *N*-dimensional space, so that the mean of the values along each dimension is zero. Yet, the two molecules are oriented differently in space. Hence, the matrix representation of such molecules differs by the rotation matrix $\mathbf{R}$:

$$\boldsymbol{A} = \boldsymbol{B}\mathbf{R} \tag{S3}$$

The general formula for the covariance matrix of $\boldsymbol{A}$ is:

$$\mathbf{cov}(\boldsymbol{A}) = \frac{1}{N}(\boldsymbol{A} - \overline{\boldsymbol{A}})^{\boldsymbol{T}}(\boldsymbol{A} - \overline{\boldsymbol{A}}) \tag{S4}$$

$\overline{\boldsymbol{A}}$ is the *M*×*N* matrix that collects the means of the columns of $\boldsymbol{A}$. To simplify the formulation, but without losing generality, the scaling factor $1/_{N}$, which is considered in our implementation of this algorithm (https://github.com/denoptim-project/HSR), can be omitted. Moreover, since the mean of the values along each dimension is zero, $\overline{\boldsymbol{A}}$ is a zero matrix, and eq S4 simplifies to:

$$\mathbf{cov}(\boldsymbol{A}) = \boldsymbol{A}^{\boldsymbol{T}}\boldsymbol{A} \tag{S5}$$

Expanding using eq S3 we obtain:

$$\mathbf{cov}(\boldsymbol{A}) = (\boldsymbol{B}\mathbf{R})^{\boldsymbol{T}}(\boldsymbol{B}\mathbf{R})$$

$$= \mathbf{R}^{\boldsymbol{T}}\boldsymbol{B}^{\boldsymbol{T}}\boldsymbol{B}\mathbf{R}$$

(S6)

Using the identity of covariance, i.e., eq S5 for matrix $\boldsymbol{B}$, we can write:

$$\mathbf{cov}(\boldsymbol{A}) = \mathbf{R}^{\boldsymbol{T}}\mathbf{cov}(\boldsymbol{B})\mathbf{R} \tag{S7}$$

The eigendecomposition of the covariance matrices for $\boldsymbol{A}$ and $\boldsymbol{B}$ yields $\boldsymbol{V_a}$ and $\boldsymbol{V_b}$, i.e., the matrices for which the i-th columns are the i-th eigenvectors of $\mathbf{cov}(\boldsymbol{A})$ and $\mathbf{cov}(\boldsymbol{B})$, respectively, and $\boldsymbol{\Lambda_a}$ and $\boldsymbol{\Lambda_b}$, i.e., the diagonal matrices for which the diagonal elements are the eigenvalues of $\mathbf{cov}(\boldsymbol{A})$ and $\mathbf{cov}(\boldsymbol{B})$, respectively:

$$\mathbf{cov}(\boldsymbol{A}) = \boldsymbol{V_a}\boldsymbol{\Lambda_a}\boldsymbol{V_a^T} \tag{S8}$$

$$\mathbf{cov}(\boldsymbol{B}) = \boldsymbol{V_b}\boldsymbol{\Lambda_b}\boldsymbol{V_b^T} \tag{S9}$$

As explained in Section S.1.1, the sign of any such eigenvector is arbitrary, and we can express this arbitrariness as:

$$\boldsymbol{V_a} = \boldsymbol{V_a^\dagger}\mathbf{D}_{\boldsymbol{a}} \tag{S10}$$

$$\boldsymbol{V}_b = \boldsymbol{V_b^\dagger}\mathbf{D}_{\boldsymbol{b}} \tag{S11}$$

where $\mathbf{D}_{\boldsymbol{a}}$ and $\mathbf{D}_{\boldsymbol{b}}$ are unknown reflection matrices. Since the arbitrary orientation is accounted for by $\mathbf{D}_{\boldsymbol{a}}$ and $\mathbf{D}_{\boldsymbol{b}}$, we can assume, without any loss of generality, that $\boldsymbol{V_a^\dagger}$ and $\boldsymbol{V_b^\dagger}$ are the eigenvector matrices with the orientations that reflect the criterion aimed for in Section S.1.1, i.e., an orientation that ensures that the score with the largest absolute value not counterbalanced by a corresponding, oppositely signed, score of value equal to within a predefined numerical tolerance is positive.

Using the above, we can now rewrite eq S8 and eq S9 as follows:

$$\mathbf{cov}(\boldsymbol{A}) = \boldsymbol{V_a^\dagger}\mathbf{D}_{\boldsymbol{a}}\boldsymbol{\Lambda_a}\mathbf{D}_{\boldsymbol{a}}^{\boldsymbol{T}}\boldsymbol{V_a^{\dagger T}} \tag{S12}$$

$$\mathbf{cov}(\boldsymbol{B}) = \boldsymbol{V_b^\dagger}\mathbf{D}_{\boldsymbol{b}}\boldsymbol{\Lambda_b}\mathbf{D}_{\boldsymbol{b}}^{\boldsymbol{T}}\boldsymbol{V_b^{\dagger T}} \tag{S13}$$

Using eq S12 and eq S13 in eq S7, we obtain:

$$\boldsymbol{V_a^\dagger}\mathbf{D}_{\boldsymbol{a}}\boldsymbol{\Lambda_a}\mathbf{D}_{\boldsymbol{a}}^{\boldsymbol{T}}\boldsymbol{V_a^{\dagger T}} = \mathbf{R}^{\boldsymbol{T}}\boldsymbol{V_b^\dagger}\mathbf{D}_{\boldsymbol{b}}\boldsymbol{\Lambda_b}\mathbf{D}_{\boldsymbol{b}}^{\boldsymbol{T}}\boldsymbol{V_b^{\dagger T}}\mathbf{R} \tag{S14}$$

Given that $\boldsymbol{\Lambda} = \mathbf{D}\boldsymbol{\Lambda}\mathbf{D}^{\boldsymbol{T}}$, we can write:

$$\boldsymbol{V_a^\dagger}\boldsymbol{\Lambda_a}\boldsymbol{V_a^{\dagger T}} = \mathbf{R}^{\boldsymbol{T}}\boldsymbol{V_b^\dagger}\boldsymbol{\Lambda_b}\boldsymbol{V_b^{\dagger T}}\mathbf{R} \tag{S15}$$

By exploiting the relation $\boldsymbol{\Lambda_a} = \boldsymbol{\Lambda_b}$, which is justified by $\mathbf{cov}(\boldsymbol{A})$ and $\mathbf{cov}(\boldsymbol{B})$ being similar matrices, i.e., $\mathbf{cov}(\boldsymbol{A}) = \mathbf{R}^{\boldsymbol{T}}\mathbf{cov}(\boldsymbol{B})\mathbf{R}$, the above equation simplifies as follows:

$$\boldsymbol{V_a^\dagger}\boldsymbol{\Lambda_a}\boldsymbol{V_a^{\dagger T}} = \mathbf{R}^{\boldsymbol{T}}\boldsymbol{V_b^\dagger}\boldsymbol{\Lambda_a}\boldsymbol{V_b^{\dagger T}}\mathbf{R} \tag{S16}$$

For convenience we group the three main terms on each side of the equation:

$$(\boldsymbol{V}_{\boldsymbol{a}}^{\dagger})(\boldsymbol{\Lambda}_{\boldsymbol{a}})(\boldsymbol{V}_{\boldsymbol{a}}^{\dagger\boldsymbol{T}}) = (\mathbf{R}^{\boldsymbol{T}}\boldsymbol{V}_{\boldsymbol{b}}^{\dagger})(\boldsymbol{\Lambda}_{\boldsymbol{a}})(\boldsymbol{V}_{\boldsymbol{b}}^{\dagger\boldsymbol{T}}\mathbf{R}) \tag{S17}$$

The central term on both sides is the eigenvalue matrix $\boldsymbol{\Lambda}_{\boldsymbol{a}}$. If we assume that the terms to the left of $\boldsymbol{\Lambda}_{\boldsymbol{a}}$ on each side are equal ($\boldsymbol{V}_{\boldsymbol{a}}^{\dagger} = \mathbf{R}^{\boldsymbol{T}}\boldsymbol{V}_{\boldsymbol{b}}^{\dagger}$), and we consider the transpose of this relation ($\boldsymbol{V}_{\boldsymbol{a}}^{\dagger\boldsymbol{T}} = \boldsymbol{V}_{\boldsymbol{b}}^{\dagger\boldsymbol{T}}\mathbf{R}$), we see that the assumption holds true because the latter relation contains the terms to the right of $\boldsymbol{\Lambda}_{\boldsymbol{a}}$ on each side eq S17, leading to

$$\boldsymbol{V}_{\boldsymbol{a}}^{\dagger} = \mathbf{R}^{\boldsymbol{T}}\boldsymbol{V}_{\boldsymbol{b}}^{\dagger} \text{ and } \boldsymbol{V}_{\boldsymbol{a}}^{\dagger\boldsymbol{T}} = \boldsymbol{V}_{\boldsymbol{b}}^{\dagger\boldsymbol{T}}\mathbf{R} \tag{S18}$$

Hence, $\boldsymbol{V}_{\boldsymbol{a}}^{\dagger}$ and $\boldsymbol{V}_{\boldsymbol{b}}^{\dagger}$ are two sets of eigenvectors differing only by a rotation.

If we were to transform the original set of coordinates, $\boldsymbol{A}$ and $\boldsymbol{B}$, into the PCs reference system, i.e., calculate the scores by applying transformation matrices $\boldsymbol{V}_{\boldsymbol{a}}$ and $\boldsymbol{V}_{\boldsymbol{b}}$, without first addressing the arbitrariness of the signs of the eigenvectors, we would obtain:

$$\boldsymbol{A}' = \mathbf{A}\boldsymbol{V}_{\boldsymbol{a}} = \boldsymbol{A}\boldsymbol{V}_{\boldsymbol{a}}^{\dagger}\mathbf{D}_{\boldsymbol{a}} = \boldsymbol{B}\mathbf{R}\boldsymbol{V}_{\boldsymbol{a}}^{\dagger}\mathbf{D}_{\boldsymbol{a}} = \boldsymbol{B}\boldsymbol{V}_{\boldsymbol{b}}^{\dagger}\mathbf{D}_{\boldsymbol{a}} \tag{S19}$$

$$\boldsymbol{B}' = \mathbf{B}\boldsymbol{V}_{\boldsymbol{b}} = \boldsymbol{B}\boldsymbol{V}_{\boldsymbol{b}}^{\dagger}\mathbf{D}_{\boldsymbol{b}} = \boldsymbol{B}\boldsymbol{V}_{\boldsymbol{b}}^{\dagger}\mathbf{D}_{\boldsymbol{b}} \tag{S20}$$

Therefore, unless $\mathbf{D}_{\boldsymbol{a}} = \mathbf{D}_{\boldsymbol{b}}$, which can only occur if, by chance, the arbitrary sign assignation is identical for the two molecules, we have that $\boldsymbol{A}' \neq \boldsymbol{B}'$. Hence, a transformation that does not addresses the arbitrariness of the signs of the eigenvectors produces two different sets of transformed coordinates for the same molecule.

Instead, if we transform using $\boldsymbol{V}_{\boldsymbol{a}}^{\dagger}$ and $\boldsymbol{V}_{\boldsymbol{b}}^{\dagger}$, we obtain:

$$\boldsymbol{A}^{\dagger} = \boldsymbol{A}\boldsymbol{V}_{\boldsymbol{a}}^{\dagger} = \boldsymbol{B}\mathbf{R}\mathbf{R}^{\boldsymbol{T}}\boldsymbol{V}_{\boldsymbol{b}}^{\dagger} = \boldsymbol{B}\boldsymbol{V}_{\boldsymbol{b}}^{\dagger} = \boldsymbol{B}^{\dagger} \tag{S21}$$

This demonstrates that two molecules with identical but differently oriented geometries will, after application of the method described in Section S.1.1, give the exact same geometry.

S.1.2. Chiral Discrimination Algorithm

Chirality is a fundamental property of some chemical objects but is not captured by the reference system described in Section S.1.1. In fact, the recipe of Section S.1.1 applied to a pair of enantiomeric collections of points (i.e., collections that are non-superimposable mirror images of each other) will generate two enantiomeric reference systems. These, in turn, will result in enantiomeric sets of reference points, identical distributions of distances, and identical fingerprints for the two enantiomers.

To enable the distinction of enantiomers using only their intrinsic PC-based reference systems, we leverage the fact that, for normalized PCs and in the absence of zero-variance dimensions, the determinant of $\boldsymbol{V}$, $det(\boldsymbol{V})$, can only be +1 or -1. In three-dimensional space, these two cases correspond to the well-known right-handed and left-handed coordinate systems, respectively. The concept of "handedness" can be generalized to higher dimensions by the sign of the reference system's determinant.[1] As a result, the PC-based reference system of two enantiomers, computed

as in Section S.1.1, are reflections of one another and the corresponding determinants have opposite sign.

These signs, moreover, reflect the convention embedded in the procedure discussed in Section S.1.1, which ensures a positive value for the largest PC score by, if necessary, changing the sign of the corresponding PC. An even number of sign changes equates to rotating the PC-based reference system and to preserving the sign of its determinant. In contrast, an odd number of changes equates to reflecting the PCs reference system and to changing the sign of the determinant of $\boldsymbol{V}$.

Since, as discussed in Section S.1.1, the sign of a PC is arbitrary, we can modify the procedure described in Section S.1.1 so that the resulting $det(\boldsymbol{V})$ is, by convention, always positive. This convention determines the handedness of the PC-based reference system and causes a pair of enantiomers to be described by the same reference system. As a result, the handedness of the reference system will, for one of the enantiomers, differ from that of the *hypershape* data points. This ensures that different fingerprints are produced for the two enantiomers.

Overall, the modified, chirality-aware, procedure for obtaining $\boldsymbol{V}$ consist of three main steps:

1. **Determinant Calculation** (line 4 in Algorithm S2)

   Compute the determinant of $\boldsymbol{V}$, $det_i = det(\boldsymbol{V})$.

2. **Orientation of PCs Based on PCA Scores** (lines 5–13 in Algorithm S2)

   The orientation of each PC (eigenvector) is chosen based on the relative PCA scores. The number of orientation changes (n_c) is recorded for the next step.

3. **Chirality Detection via Determinant Constraint** (lines 14–16 in Algorithm S2)

   The parity of n_c tells whether the determinant of $\boldsymbol{V}$ has changed. Therefore, the determinant of the new matrix of eigenvectors is computed as $det_f = det_i \cdot (-1)^{n_c}$. If $det_f = -1$, a PC reflection is applied to ensure that $det(\boldsymbol{V})$ is equal to +1.

Algorithm S2. Chirality Distinction: PCA-based Orientation of Principal Components.

1- ***Input:*** *N-dimensional matrix of coordinates* $\boldsymbol{A}$

2- *Covariance matrix of* $\boldsymbol{A}$: $\boldsymbol{cov}(\boldsymbol{A}) = \frac{1}{N}(\boldsymbol{A} - \overline{\boldsymbol{A}})^{\boldsymbol{T}}(\boldsymbol{A} - \overline{\boldsymbol{A}})$

3- *Principal Components* $\boldsymbol{V}$*: Eigendecomposition(*$\boldsymbol{cov}(\boldsymbol{A})$*)*

COMPUTE DETERMINANT OF V:

4- $det_i = det(\boldsymbol{V})$

ORIENT PRINCIPAL COMPONENTS BASED ON PCA SCORES:

5- *Projection matrix/PCA scores* $\boldsymbol{P}$: $\boldsymbol{P} = \boldsymbol{AV}$

6- *Initialize* $n = 0$

7- *For each column j in* $\boldsymbol{P}$*:*

8- | *Find index of maximum PCA score in column j:* $k = argmax(\boldsymbol{p}_{[:,j]})$

9- | | *If* $p_{k,j} < 0$*:*

10- | | | *Flip the orientation of the j-th eigenvector in* $\boldsymbol{V}$: $\boldsymbol{v}'_{[:,j]} = -\boldsymbol{v}_{[:,j]}$

Increment n_c: $n_c = n_c + 1$

else $p_{k,j} > 0$

$\boldsymbol{v}'_{[:,\boldsymbol{j}]} = \boldsymbol{v}_{[:,\boldsymbol{j}]}$

DETECT CHIRALITY VIA DETERMINANT CONSTRAINT:

If $det_f = det_i \cdot (-1)^{n_c} < 0$:

Reflection matrix $\boldsymbol{D_1}^a$: $\boldsymbol{D_1} = \begin{bmatrix} -\mathbf{1} & \mathbf{0} & \dots \\ \mathbf{0} & \mathbf{1} & \dots \\ \vdots & \vdots & \ddots \end{bmatrix}$

Update $\boldsymbol{V}'$: $\boldsymbol{V}'' = \boldsymbol{V}'\boldsymbol{D_1}$

Output: $\boldsymbol{V}''$

---

[a]The reflection matrix $\mathbf{D_1}$, used to constrain the determinant, can act on any eigenvector. In the above description, $\mathbf{D_1}$ acts on the first eigenvector. In the software implementation (https://github.com/denoptim-project/HSR) $\mathbf{D_1}$ is applied to the eigenvector for which the relative PCA scores produce the greatest skewness.

S.1.2.1. Demonstration

To demonstrate how the above-described method distinguishes enantiomers while still handling identical molecules, we present a stepwise demonstration of how the method is applied in the two cases.

S.1.2.1.1 Identical Molecules

Consider two identical molecules, *A* and *B*, consisting of *M* atoms, each represented by 3D Cartesian coordinates plus any number of atomic features to compose an *N*-dimensional representation. These coordinates are collected in two matrices *M*×*N*, $\boldsymbol{A}$ and $\boldsymbol{B}$. The molecules have identical geometry, and their geometrical center coincides with the origin of the *N*-dimensional space, so that the mean of the values along each dimension is zero, but the two molecules can be oriented differently in space. So, the matrix representations differ by the rotation matrix **R**:

$$\boldsymbol{A} = \boldsymbol{B}\mathbf{R} \tag{S22}$$

The covariance matrix of $\boldsymbol{A}$, denoted as $\mathbf{cov}(\boldsymbol{A})$, is given by (the same consideration done for eq S5 are valid here: centered coordinates and omission of the factor $1/_N$):

$$\mathbf{cov}(\boldsymbol{A}) = \boldsymbol{A}^T\boldsymbol{A} \tag{S23}$$

Expanding eq S23 using eq S22 we obtain:

$$\mathbf{cov}(\boldsymbol{A}) = (\boldsymbol{B}\mathbf{R})^T(\boldsymbol{B}\mathbf{R}) \\ = \mathbf{R}^T\boldsymbol{B}^T\boldsymbol{B}\mathbf{R} \tag{S24}$$

Using the definition of covariance of $\boldsymbol{B}$, which is analogous to eq S23, we can write:

$$\mathbf{cov}(\boldsymbol{A}) = \mathbf{R}^{T}\mathbf{cov}(\boldsymbol{B})\mathbf{R} \tag{S25}$$

Given the eigen-decomposition of the covariance matrices for $\boldsymbol{A}$ and $\boldsymbol{B}$:

$$\mathbf{cov}(\boldsymbol{A}) = \boldsymbol{V_a}\boldsymbol{\Lambda_a}\boldsymbol{V_a^T} \tag{S26}$$

$$\mathbf{cov}(\boldsymbol{B}) = \boldsymbol{V_b}\boldsymbol{\Lambda_b}\boldsymbol{V_b^T} \tag{S27}$$

and accounting for the arbitrariness of eigenvectors' sign, as shown in Section S.1.1:

$$\mathbf{cov}(\boldsymbol{A}) = \boldsymbol{V_a^{\dagger}}\mathbf{D}_{\boldsymbol{a}}\boldsymbol{\Lambda_a}\mathbf{D}_{\boldsymbol{a}}^{\boldsymbol{T}}\boldsymbol{V_a^{\dagger T}} \tag{S28}$$

$$\mathbf{cov}(\boldsymbol{B}) = \boldsymbol{V_b^{\dagger}}\mathbf{D}_{\boldsymbol{b}}\boldsymbol{\Lambda_b}\mathbf{D}_{\boldsymbol{b}}^{\boldsymbol{T}}\boldsymbol{V_b^{\dagger T}} \tag{S29}$$

We then apply the chiral discrimination algorithm described in Section S.1.2.

1. **Determinant Calculation**

   The determinants of the eigenvector matrices $\boldsymbol{V_a}$ and $\boldsymbol{V_b}$ are computed.

2. **Orientation of PCs Based on PCA Scores**

   Given that molecules *A* and *B* have the same geometry, the respective PCA scores will produce PCs reflections leading to two reference systems with the same determinant. As shown in Section S.1.1, this operation will produce the two sets of eigenvectors $\boldsymbol{V_a^{\dagger}}$ and $\boldsymbol{V_b^{\dagger}}$.

3. **Chirality Detection by Determinant Imposition**

   Having the same determinants, the two sets of eigenvectors, $\boldsymbol{V_a^{\dagger}}$ and $\boldsymbol{V_b^{\dagger}}$**,** will undergo the same transformation:

   If $det(\boldsymbol{V_a^{\dagger}})$ and $det(\boldsymbol{V_b^{\dagger}})$ are equal to +1 (i.e., no transformation):

$$\boldsymbol{V'_a} = \boldsymbol{V_a^{\dagger}} \tag{S30}$$

$$\boldsymbol{V'_b} = \boldsymbol{V_b^{\dagger}} \tag{S31}$$

   And if $det(\boldsymbol{V_a^{\dagger}})$ and $det(\boldsymbol{V_b^{\dagger}})$ are equal to –1 (i.e., reflection of the same PC):

$$\boldsymbol{V'_a} = \boldsymbol{V_a^{\dagger}}\mathbf{D} \tag{S32}$$

$$\boldsymbol{V'_b} = \boldsymbol{V_b^{\dagger}}\mathbf{D} \tag{S33}$$

In the first case, where $det(\boldsymbol{V_a^{\dagger}})$ and $det(\boldsymbol{V_b^{\dagger}})$ are equal to +1, both matrices $\boldsymbol{V_a^{\dagger}}$ and $\boldsymbol{V_a^{\dagger}}$ remain unchanged and hence:

$$\boldsymbol{V_a^{\dagger}} = \mathbf{R}^{\boldsymbol{T}}\boldsymbol{V_b^{\dagger}} \Rightarrow \boldsymbol{V'_a} = \mathbf{R}^{\boldsymbol{T}}\boldsymbol{V'_b} \tag{S34}$$

And, in the second case, where $det(\boldsymbol{V}_{\boldsymbol{a}}^{\dagger})$ and $det(\boldsymbol{V}_{\boldsymbol{b}}^{\dagger})$ are equal to –1:

$$\boldsymbol{V}_{\boldsymbol{a}}^{\dagger} = \mathbf{R}^{\boldsymbol{T}}\boldsymbol{V}_{\boldsymbol{b}}^{\dagger}$$

$$\Rightarrow \boldsymbol{V}'_{\boldsymbol{a}}\mathbf{D} = \mathbf{R}^{\boldsymbol{T}}\boldsymbol{V}'_{\boldsymbol{b}}\mathbf{D}$$

$$\Rightarrow \boldsymbol{V}'_{\boldsymbol{a}} = \mathbf{R}^{\boldsymbol{T}}\boldsymbol{V}'_{\boldsymbol{b}}$$

(S35)

Therefore, the relation $\boldsymbol{V}'_{\boldsymbol{a}} = \mathbf{R}^{\boldsymbol{T}}\boldsymbol{V}'_{\boldsymbol{b}}$ is preserved in both cases.

Finally, the matrix representation of each molecule in the respective reference systems defined by our method can be obtained by transforming the original matrices with the corresponding transformation matrix:

$$\boldsymbol{A}' = \boldsymbol{A}\boldsymbol{V}'_{\boldsymbol{a}} \quad \text{(S36)}$$

$$\boldsymbol{B}' = \boldsymbol{B}\boldsymbol{V}'_{\boldsymbol{b}} \quad \text{(S37)}$$

Using the eq S36 for $\boldsymbol{A}$ and eq S34 for $\boldsymbol{V}'_{\boldsymbol{a}}$ we can show that:

$$\boldsymbol{A}' = \boldsymbol{A}\boldsymbol{V}'_{\boldsymbol{a}}$$

$$= \boldsymbol{B}\mathbf{R}\mathbf{R}^{\boldsymbol{T}}\boldsymbol{V}'_{\boldsymbol{b}}$$

$$= \boldsymbol{B}\boldsymbol{V}'_{\boldsymbol{b}}$$

$$= \boldsymbol{B}'$$

(S38)

Hence, irrespectively on the parity of n_a and n_b, the transformation of $\boldsymbol{A}$ and $\boldsymbol{B}$ into the PCs reference system defined according to the method described in Section S.1.2, yields $\boldsymbol{A}' = \boldsymbol{B}'$ demonstrating that the two identical, yet differently oriented molecules will be represented by the same matrix in the PCs reference system and, therefore, will produce the same fingerprint.

S.1.2.1.2 Enantiomers

We now consider two enantiomers *A* and *B* represented by their respective *N*-dimensional coordinates' matrices *M*×*N*, $\boldsymbol{A}$ and $\boldsymbol{B}$, where M is the number of atoms. Since enantiomers are non-superimposable mirror images, each enantiomer can be expressed as the reflection of the other. If we also account for different orientations (while still centered coordinates, i.e., the mean of the values along each dimension is zero), as in the identical molecules case of Section S.1.2.1.1, we obtain that an enantiomer can be expressed as the reflection and rotation of the other:

$$\boldsymbol{A} = \boldsymbol{B}\mathbf{M}\mathbf{R} \quad \text{(S39)}$$

So, the two molecules differ by a rotation matrix **R** and a reflection matrix **M** (mirror).

The covariance matrix for $\boldsymbol{A}$, denoted $\mathbf{cov}(\boldsymbol{A})$, (as in eq S5 and eq S23) is given by:

$$\mathbf{cov}(\boldsymbol{A}) = \boldsymbol{A}^{\boldsymbol{T}}\boldsymbol{A} \tag{S40}$$

Expanding using the given relation:

$$\begin{aligned}\mathbf{cov}(\boldsymbol{A}) &= (\boldsymbol{B}\mathbf{MR})^{\boldsymbol{T}}(\boldsymbol{B}\mathbf{MR}) \\ &= \mathbf{R}^{\boldsymbol{T}}\mathbf{M}^{\boldsymbol{T}}\boldsymbol{B}^{\boldsymbol{T}}\boldsymbol{B}\mathbf{MR}\end{aligned} \tag{S41}$$

Using the identity of covariance, we can write:

$$\mathbf{cov}(\boldsymbol{A}) = \mathbf{R}^{\boldsymbol{T}}\mathbf{M}^{\boldsymbol{T}}\mathbf{cov}(\boldsymbol{B})\mathbf{MR} \tag{S42}$$

Given the eigendecomposition of the covariance matrices for $\boldsymbol{A}$ and $\boldsymbol{B}$:

$$\mathbf{cov}(\boldsymbol{A}) = \boldsymbol{V_a}\boldsymbol{\Lambda_a}\boldsymbol{V_a^T} \tag{S43}$$

$$\mathbf{cov}(\boldsymbol{B}) = \boldsymbol{V_b}\boldsymbol{\Lambda_b}\boldsymbol{V_b^T} \tag{S44}$$

and accounting for the arbitrariness of eigenvectors' sign, as shown in Section S.1.1:

$$\mathbf{cov}(\boldsymbol{A}) = \boldsymbol{V_a^\dagger}\mathbf{D}_{\boldsymbol{a}}\boldsymbol{\Lambda_a}\mathbf{D}_{\boldsymbol{a}}^{\boldsymbol{T}}\boldsymbol{V_a^{\dagger T}} \tag{S45}$$

$$\mathbf{cov}(\boldsymbol{B}) = \boldsymbol{V_b^\dagger}\mathbf{D}_{\boldsymbol{b}}\boldsymbol{\Lambda_b}\mathbf{D}_{\boldsymbol{b}}^{\boldsymbol{T}}\boldsymbol{V_b^{\dagger T}} \tag{S46}$$

In this case, $\boldsymbol{V_a^\dagger}$ and $\boldsymbol{V_b^\dagger}$ are the two sets of eigenvectors differing only by a rotation and a reflection (mirroring of the two enantiomers), which can be obtained as shown in eq S18:

$$\begin{aligned}\boldsymbol{V_a^\dagger}\mathbf{D}_{\boldsymbol{a}}\boldsymbol{\Lambda_a}\mathbf{D}_{\boldsymbol{a}}^{\boldsymbol{T}}\boldsymbol{V_a^{\dagger T}} &= \mathbf{R}^{\boldsymbol{T}}\mathbf{M}^{\boldsymbol{T}}\boldsymbol{V_b^\dagger}\mathbf{D}_{\boldsymbol{b}}\boldsymbol{\Lambda_b}\mathbf{D}_{\boldsymbol{b}}^{\boldsymbol{T}}\boldsymbol{V_b^{\dagger T}}\mathbf{MR} \\ \Rightarrow \boldsymbol{V_a^\dagger}\boldsymbol{\Lambda_a}\boldsymbol{V_a^{\dagger T}} &= \mathbf{R}^{\boldsymbol{T}}\mathbf{M}^{\boldsymbol{T}}\boldsymbol{V_b^\dagger}\boldsymbol{\Lambda_b}\boldsymbol{V_b^{\dagger T}}\mathbf{MR} \\ &= \mathbf{R}^{\boldsymbol{T}}\mathbf{M}^{\boldsymbol{T}}\boldsymbol{V_b^\dagger}\boldsymbol{\Lambda_a}\boldsymbol{V_b^{\dagger T}}\mathbf{MR} \\ &\Rightarrow \boldsymbol{V_a^\dagger} = \mathbf{R}^{\boldsymbol{T}}\mathbf{M}^{\boldsymbol{T}}\boldsymbol{V_b^\dagger}\end{aligned} \tag{S47}$$

We then apply the chiral discrimination algorithm described in Section S.1.2.

1. **Determinant Calculation**

   The determinants of the eigenvector matrices $\boldsymbol{V_a}$ and $\boldsymbol{V_b}$ are computed.

2. **Orientation of PCs Based on PCA Scores**

Enantiomers are separated by a reflection ($\mathbf{M}$). Hence, their PCs reference systems, constructed upon their PCA scores, have opposite determinants. As shown in Section S.1.1, this operation will produce the two sets of eigenvectors $\boldsymbol{V}_{\boldsymbol{a}}^{\dagger}$ and $\boldsymbol{V}_{\boldsymbol{b}}^{\dagger}$.

3. **Chirality Detection by Determinant Imposition**

   Since $\boldsymbol{V}_{\boldsymbol{a}}^{\dagger}$ and $\boldsymbol{V}_{\boldsymbol{b}}^{\dagger}$ have determinants with opposite sign, the two sets of eigenvectors will now undergo a different operation, i.e., the one that has a determinant equal to -1 will undergo an extra reflection. For example, we consider the case where $det(\boldsymbol{V}_{\boldsymbol{a}}^{\dagger}) = 1$ and $det(\boldsymbol{V}_{\boldsymbol{b}}^{\dagger}) = -1$:

$$\boldsymbol{V}_{\boldsymbol{a}}' = \boldsymbol{V}_{\boldsymbol{a}}^{\dagger} \quad \text{(S48)}$$

$$\boldsymbol{V}_{\boldsymbol{b}}' = \boldsymbol{V}_{\boldsymbol{b}}^{\dagger}\mathbf{S} \quad \text{(S49)}$$

With eq S49, we obtain $det(\boldsymbol{V}_{\boldsymbol{b}}') = 1$ by performing an additional reflection only on $\boldsymbol{V}_{\boldsymbol{b}}^{\dagger}$. For the sake of simplicity, but without losing generality, we can assume that such additional reflection is equal to the initial reflection $\mathbf{M}$ ($\mathbf{S} = \mathbf{M}$), and since $\boldsymbol{V}_{\boldsymbol{b}}^{\dagger}$ is a square matrix and $\mathbf{M}^{\boldsymbol{T}}$ is a diagonal matrix:

$$\boldsymbol{V}_{\boldsymbol{b}}' = \boldsymbol{V}_{\boldsymbol{b}}^{\dagger}\mathbf{M}^{\boldsymbol{T}} = \mathbf{M}^{\boldsymbol{T}}\boldsymbol{V}_{\boldsymbol{b}}^{\dagger} \quad \text{(S50)}$$

$$\mathbf{M}\boldsymbol{V}_{\boldsymbol{b}}' = \boldsymbol{V}_{\boldsymbol{b}}^{*} \quad \text{(S51)}$$

We then use eq S51 and eq S48 to obtain the relation:

$$\boldsymbol{V}_{\boldsymbol{a}}^{\dagger} = \mathbf{R}^{\boldsymbol{T}}\mathbf{M}^{\boldsymbol{T}}\boldsymbol{V}_{\boldsymbol{b}}^{\dagger}$$

$$\Rightarrow \boldsymbol{V}_{\boldsymbol{a}}' = \mathbf{R}^{\boldsymbol{T}}\mathbf{M}^{\boldsymbol{T}}\boldsymbol{M}\boldsymbol{V}_{\boldsymbol{b}}'$$

$$\Rightarrow \boldsymbol{V}_{\boldsymbol{a}}' = \mathbf{R}^{\boldsymbol{T}}\boldsymbol{V}_{\boldsymbol{b}}'$$

(S52)

Therefore, the eigenvector matrices are separated by only a rotation:

$$\boldsymbol{V}_{\boldsymbol{a}}' = \mathbf{R}^{\boldsymbol{T}}\boldsymbol{V}_{\boldsymbol{b}}' \quad \text{(S53)}$$

Which leads to the transformation:

$$\begin{aligned} \boldsymbol{A}' &= \boldsymbol{A}\boldsymbol{V}_{\boldsymbol{a}}' \\ &= \boldsymbol{B}\mathbf{M}\mathbf{R}\mathbf{R}^{\boldsymbol{T}}\boldsymbol{V}_{\boldsymbol{b}}' \\ &= \mathbf{B}\mathbf{M}\boldsymbol{V}_{\boldsymbol{b}}' \\ &= \boldsymbol{B}\boldsymbol{V}_{\boldsymbol{b}}'\mathbf{M} \\ &= \boldsymbol{B}'\mathbf{M} \end{aligned}$$

$$(S54)$$

Since $\boldsymbol{A}' = \boldsymbol{B}'\mathbf{M}$, the matrix representations of the two enantiomers still differ by reflection upon transformation into their respective PCs reference systems. Therefore, the distribution of distance from the reference points located along the PCs will be different for the two enantiomers leading to a different fingerprint for each of the two molecules, thereby demonstrating the effectiveness of the method described in Section S.1.2 in distinguishing enantiomers.

S.2. Numerical Example

In this section, we illustrate the method's functionality by using a straightforward numerical example with calculations that can be easily performed manually. The numerical results can also be verified with the corresponding script made available in Zenodo (https://doi.org/10.5281/zenodo.14631654).

This example consists of computing the similarity between two theoretical diatomic molecules: (*A*) H–Li, (*B*) H–He. Both molecules are assumed to have the same bond length of 2 Å. In the following, we manually create fingerprints for these molecules and use them to calculate the HSR score. The chiral discrimination method described in Section S.1.2 is not applied in this example.

Although the software uses a six-dimensional (6D) representation, our manual calculations will focus on only two dimensions—the non-redundant spatial coordinate corresponding to the sole internal coordinate of a diatomic molecule (i.e., the bond length) and the proton number—because these are the only dimensions with non-zero variance for these molecules. Hence, a bi-dimensional representation suffices to completely characterize the two molecules.

S.2.1. Determination of the Fingerprint for Molecule *A*

The initial coordinates of molecule *A* are collected in the matrix $\boldsymbol{A}$. In 6D, these coordinates are:

$$\boldsymbol{A} = \begin{bmatrix} -1 & 0 & 0 & 1 & 0 & 0 \\ 1 & 0 & 0 & 3 & 0 & 0 \end{bmatrix} \tag{S55}$$

Where the first three columns correspond to the atomic Cartesian coordinates, the fourth to the number of protons (without the square root), the fifth to the number of neutrons (compared to the number of neutrons of the most common isotope), and the sixth to the formal charge.

After centering, these coordinates become:

$$\boldsymbol{A} = \begin{bmatrix} -1 & 0 & 0 & -1 & 0 & 0 \\ 1 & 0 & 0 & 1 & 0 & 0 \end{bmatrix} \tag{S56}$$

As mentioned above, given that only two dimensions display non-zero variance, our analysis can be simplified by focusing solely on these two dimensions. Hence, molecule *A*'s matrix of coordinates $\boldsymbol{A}$ can be represented as:

$$\boldsymbol{A} = \begin{bmatrix} -1 & -1 \\ 1 & 1 \end{bmatrix} \tag{S57}$$

Covariance Matrix. The covariance matrix for $\boldsymbol{A}$ is calculated as:

$$
\begin{aligned}
\boldsymbol{covA} &= \frac{1}{2}\boldsymbol{A^T A} \\
&= \frac{1}{2}\begin{bmatrix} -1 & 1 \\ -1 & 1 \end{bmatrix}\begin{bmatrix} -1 & -1 \\ 1 & 1 \end{bmatrix} \\
&= \frac{1}{2}\begin{bmatrix} (-1 \cdot -1) + (1 \cdot 1) & (-1 \cdot -1) + (1 \cdot 1) \\ (-1 \cdot -1) + (1 \cdot 1) & (-1 \cdot -1) + (1 \cdot 1) \end{bmatrix} \\
&= \frac{1}{2}\begin{bmatrix} 2 & 2 \\ 2 & 2 \end{bmatrix} \\
&= \begin{bmatrix} 1 & 1 \\ 1 & 1 \end{bmatrix}
\end{aligned}
\tag{S58}
$$

Eigendecomposition. The eigenvectors and eigenvalues are determined as follows:

$$
\begin{aligned}
det(\boldsymbol{covA} - \lambda \boldsymbol{I}) &= det\left(\begin{bmatrix} 1-\lambda & 1 \\ 1 & 1-\lambda \end{bmatrix}\right) \\
&= (1-\lambda)(1-\lambda) - 1 \\
&= \lambda^2 - 2\lambda
\end{aligned}
\tag{S59}
$$

Setting the determinant to zero gives the secular equation:

$$\lambda^2 - 2\lambda = 0 \tag{S60}$$

Solving for λ we find the eigenvalues:

$$\lambda_1 = 2 \tag{S61}$$

$$\lambda_2 = 0 \tag{S62}$$

Determination of eigenvectors:

For λ_1:

$$\begin{bmatrix} -1 & 1 \\ 1 & -1 \end{bmatrix}\boldsymbol{v}_1 = 0 \tag{S63}$$

From which, the system of equations is:

$$\begin{cases} -v_{1,1} + v_{1,2} = 0 \\ v_{1,1} - v_{1,2} = 0 \end{cases} \tag{S64}$$

Solving, we find:

$$\boldsymbol{v}_1 = \begin{bmatrix} 1 \\ 1 \end{bmatrix} \tag{S65}$$

For λ_2:

$$\begin{bmatrix} 1 & 1 \\ 1 & 1 \end{bmatrix}\boldsymbol{v}_2 = 0 \tag{S66}$$

From which we get the system of equations:

$$\begin{cases} v_{2,1} + v_{2,2} = 0 \\ v_{2,1} + v_{2,2} = 0 \end{cases} \quad \text{(S67)}$$

From eq S67, we get $v_{2,1} = -v_{2,2}$.

Taking $v_{2,1} = 1$, we get $v_{2,2} = -1$. Thus, the eigenvector is:

$$\boldsymbol{v}_2 = \begin{bmatrix} -1 \\ 1 \end{bmatrix} \quad \text{(S68)}$$

To normalize, we divide each component by its magnitude.

The magnitude of both vectors is $\sqrt{(-1)^2 + 1^2} = \sqrt{2}$.

Here are shown the eigenvalues and normalized eigenvectors:

$$\lambda_1 = 2, \qquad \lambda_2 = 0, \qquad \boldsymbol{V_a} = \begin{bmatrix} \frac{1}{\sqrt{2}} & -\frac{1}{\sqrt{2}} \\ \frac{1}{\sqrt{2}} & \frac{1}{\sqrt{2}} \end{bmatrix}$$

It is important to remember that we focused on the 2D representation. However, this simplification is not adopted in our Python implementation. Therefore, the results from the latter implementation consist of six 6D eigenvectors, only two of which have more than one non-zero component. The other eigenvectors correspond to the dimensions where the molecule's coordinates are 0, hence, for now, they are ignored in the 2D case for the sake of clarity.

Projections/PCA Scores. Here, the algorithm performs the operation $\boldsymbol{P} = \boldsymbol{A}\boldsymbol{V_a}$ to determine the orientation of the eigenvectors. We compute one eigenvector at the time.

For $\boldsymbol{v}_1$:

$$\begin{bmatrix} -1 & -1 \\ 1 & 1 \end{bmatrix} \frac{1}{\sqrt{2}} \begin{bmatrix} 1 \\ 1 \end{bmatrix} = \frac{1}{\sqrt{2}} \begin{bmatrix} -1 - 1 \\ 1 + 1 \end{bmatrix}$$
$$= \frac{1}{\sqrt{2}} \begin{bmatrix} -2 \\ 2 \end{bmatrix}$$
$$= \begin{bmatrix} -\sqrt{2} \\ \sqrt{2} \end{bmatrix}$$

(S69)

There are only two PCA scores and they have the same absolute value, so the sign of the eigenvector is irrelevant. Hence the sign of the eigenvector is not changed.

For $\boldsymbol{v}_2$:

$$\begin{bmatrix} -1 & -1 \\ 1 & 1 \end{bmatrix} \frac{1}{\sqrt{2}} \begin{bmatrix} -1 \\ 1 \end{bmatrix} = \frac{1}{\sqrt{2}} \begin{bmatrix} 1 - 1 \\ -1 + 1 \end{bmatrix}$$

$$= \frac{1}{\sqrt{2}} \begin{bmatrix} 0 \\ 0 \end{bmatrix}$$

$$= \begin{bmatrix} 0 \\ 0 \end{bmatrix}$$

(S70)

As before, the sign of the eigenvector is not changed.

So, the matrix with sign-adjusted eigenvectors is:

$$\boldsymbol{V_a} = \begin{bmatrix} \frac{1}{\sqrt{2}} & -\frac{1}{\sqrt{2}} \\ \frac{1}{\sqrt{2}} & \frac{1}{\sqrt{2}} \end{bmatrix} \quad \text{(S71)}$$

Transformation. The coordinates of molecule *A* can be defined into the canonical basis of the PC space as $\boldsymbol{A'}$ (PCA scores).

$$\begin{aligned} \boldsymbol{A'} &= \boldsymbol{AV_a} \\ &= \begin{bmatrix} -1 & -1 \\ 1 & 1 \end{bmatrix} \frac{1}{\sqrt{2}} \begin{bmatrix} 1 & -1 \\ 1 & 1 \end{bmatrix} \\ &= \frac{1}{\sqrt{2}} \begin{bmatrix} -1-1 & 1-1 \\ 1+1 & -1+1 \end{bmatrix} \\ &= \frac{1}{\sqrt{2}} \begin{bmatrix} -2 & 0 \\ 2 & 0 \end{bmatrix} \\ &= \begin{bmatrix} -\sqrt{2} & 0 \\ \sqrt{2} & 0 \end{bmatrix} \end{aligned}$$

(S72)

Reference Points. As explained in the main paper, there are *N*+1 reference points: the origin of the PC space (geometrical center) and one reference point located along each PC. The distances of each point from the origin can be customized in our implementation, but by default each point is placed at a distance from the origin equal to the largest coordinate along that PC (largest score on that PC). When the maximum distance is zero (zero variance PC), like $\boldsymbol{v}_2$ (PC_2) in this example, the reference point is placed at a distance of 1 unit from the origin.

In order to obtain reference points with the same dimensionality reported by the script available in Zenodo (https://doi.org/10.5281/zenodo.14631654), we now return to the 6D representation. The seven resulting reference points are:

$$ref_1 = [0, 0, 0, 0, 0, 0]$$

$$ref_2 = [1.414, 0, 0, 0, 0, 0]$$

$$ref_3 = [0, 1, 0, 0, 0, 0]$$

$$ref_4 = [0, 0, 1, 0, 0, 0]$$

$$ref_5 = [0, 0, 0, 1, 0, 0]$$

$$ref_6 = [0, 0, 0, 0, 1, 0]$$

$ref_7 = [0, 0, 0, 0, 0, 1]$

For comparison with the log of the script eq S73 shows the matrix that collects the coordinates of the above reference points.

$$\begin{bmatrix} 0 & 0 & 0 & 0 & 0 & 0 \\ 1.414 & 0 & 0 & 0 & 0 & 0 \\ 0 & 1 & 0 & 0 & 0 & 0 \\ 0 & 0 & 1 & 0 & 0 & 0 \\ 0 & 0 & 0 & 1 & 0 & 0 \\ 0 & 0 & 0 & 0 & 1 & 0 \\ 0 & 0 & 0 & 0 & 0 & 1 \end{bmatrix} \quad \text{(S73)}$$

Distances. The list of Euclidean distances is computed from each of the two atoms to each reference point:

$d_{ref1} = (1.414, 1.414)$

$d_{ref2} = (2.828, 0)$

$d_{ref3} = (1.73, 1.73)$

$d_{ref4} = (1.73, 1.73)$

$d_{ref5} = (1.73, 1.73)$

$d_{ref6} = (1.73, 1.73)$

$d_{ref7} = (1.73, 1.73)$

Moments. The first three statistical moments are computed for each distance distribution:

$moments_1 = (1.414, 0, 0)$

$moments_2 = (1.414, 1.414, 0)$

$moments_3 = (1.732, 0, 0)$

$moments_4 = (1.732, 0, 0)$

$moments_5 = (1.732, 0, 0)$

$moments_6 = (1.732, 0, 0)$

$moments_7 = (1.732, 0, 0)$

Fingerprint. The moments are finally collected in the fingerprint of molecule A:

$\boldsymbol{f_A} = [1.414, 0, 0, 1.414, 1.414, 0, 1.732, 0, 0, 1.732, 0, 0, 1.732, 0, 0, 1.732, 0, 0, 1.732, 0, 0]$

S.2.2. Determination of the Fingerprint for Molecule *B*

As for the previous case, the initial coordinates of molecule B are collected in the matrix ***B*** which in 6D are:

$$\boldsymbol{B} = \begin{bmatrix} -1 & 0 & 0 & 1 & 0 & 0 \\ 1 & 0 & 0 & 2 & 0 & 0 \end{bmatrix} \quad \text{(S74)}$$

Which after centering them, become:

$$\boldsymbol{B} = \begin{bmatrix} -1 & 0 & 0 & -0.5 & 0 & 0 \\ 1 & 0 & 0 & 0.5 & 0 & 0 \end{bmatrix} \tag{S75}$$

And by ignoring the coordinates with only zero values molecule $\boldsymbol{B}$'s coordinates can be reduced to:

$$\boldsymbol{B} = \begin{bmatrix} -1 & -0.5 \\ 1 & 0.5 \end{bmatrix} \tag{S76}$$

Covariance Matrix. The covariance matrix for $\boldsymbol{B}$ is calculated as:

$$\begin{aligned} \boldsymbol{covB} &= \frac{1}{2}\boldsymbol{B^T B} \\ &= \frac{1}{2}\begin{bmatrix} -1 & 1 \\ -0.5 & 0.5 \end{bmatrix}\begin{bmatrix} -1 & -0.5 \\ 1 & 0.5 \end{bmatrix} \\ &= \frac{1}{2}\begin{bmatrix} (-1\cdot -1) + (1\cdot 1) & (-1\cdot -0.5) + (1\cdot 0.5) \\ (-0.5\cdot -1) + (0.5\cdot 1) & (-0.5\cdot -0.5) + (0.5\cdot 0.5) \end{bmatrix} \\ &= \frac{1}{2}\begin{bmatrix} 2 & 1 \\ 1 & 0.5 \end{bmatrix} \\ &= \begin{bmatrix} 1 & 0.5 \\ 0.5 & 0.25 \end{bmatrix} \end{aligned} \tag{S77}$$

Eigendecomposition. The eigenvectors and eigenvalues are determined as follows:

$$\begin{aligned} det(\boldsymbol{covA} - \lambda \boldsymbol{I}) &= det\left(\begin{bmatrix} 1-\lambda & 1 \\ 1 & 1-\lambda \end{bmatrix}\right) \\ &= (1-\lambda)(1-\lambda) - 1 \\ &= \lambda^2 - 2\lambda \end{aligned} \tag{S78}$$

Setting the determinant to zero gives the secular equation:

$$\lambda^2 - 1.25\lambda = 0 \tag{S79}$$

Solving for λ we find the eigenvalues:

$$\lambda_1 = 1.25 \tag{S80}$$

$$\lambda_2 = 0 \tag{S81}$$

Eigenvectors:

For λ_1:

$$\begin{bmatrix} -0.25 & 0.5 \\ 0.5 & -1 \end{bmatrix}\boldsymbol{v}_1 = 0 \tag{S82}$$

From which, the system of equations is:

$$\begin{cases} -0.25v_{1,1} + 0.5v_{1,2} = 0 \\ 0.5v_{1,1} - v_{1,2} = 0 \end{cases} \tag{S83}$$

Solving and normalizing as shown before, we find:

$$\boldsymbol{v}_1 = \frac{1}{\sqrt{5}}\begin{bmatrix}2\\1\end{bmatrix} = \begin{bmatrix}0.8944\\0.4472\end{bmatrix} \tag{S84}$$

For λ_2:

$$\begin{bmatrix}1 & 0.5\\0.5 & 0.25\end{bmatrix}\boldsymbol{v}_2 = 0 \tag{S85}$$

From which we get the system of equations:

$$\begin{cases} v_{2,1} + 0.5v_{2,2} = 0\\ 0.5v_{2,1} - 0.25v_{2,2} = 0\end{cases} \tag{S86}$$

Solving and normalizing as shown before, we find:

$$\boldsymbol{v}_2 = \frac{1}{\sqrt{5}}\begin{bmatrix}-1\\2\end{bmatrix} = \begin{bmatrix}-0.4472\\0.8944\end{bmatrix} \tag{S87}$$

Here are shown the eigenvalues and normalized eigenvectors:

$$\lambda_1 = 1.25, \qquad \lambda_2 = 0, \qquad \boldsymbol{V_b} = \begin{bmatrix}0.8944 & -0.4472\\0.4472 & 0.8944\end{bmatrix}$$

Projections/PCA scores. Here, the operation $\boldsymbol{P} = \boldsymbol{B}\boldsymbol{V_b}$ is performed to determine the orientation of the eigenvectors. We compute one eigenvector at the time.

For $\boldsymbol{v}_1$:

$$\begin{aligned}\begin{bmatrix}-1 & -0.5\\1 & 0.5\end{bmatrix}\frac{1}{\sqrt{5}}\begin{bmatrix}2\\1\end{bmatrix} &= \frac{1}{\sqrt{5}}\begin{bmatrix}-2-0.5\\-2+0.5\end{bmatrix}\\ &= \frac{1}{\sqrt{5}}\begin{bmatrix}-2.5\\2.5\end{bmatrix}\\ &= \begin{bmatrix}1.118\\1.118\end{bmatrix}\end{aligned} \tag{S88}$$

The two PCA scores have the same value; hence the sign of the eigenvector is not changed.

For $\boldsymbol{v}_2$:

$$\begin{aligned}\begin{bmatrix}-1 & -0.5\\1 & 0.5\end{bmatrix}\frac{1}{\sqrt{5}}\begin{bmatrix}-1\\2\end{bmatrix} &= \frac{1}{\sqrt{5}}\begin{bmatrix}1-1\\-1+1\end{bmatrix}\\ &= \frac{1}{\sqrt{5}}\begin{bmatrix}0\\0\end{bmatrix}\end{aligned}$$

$$= \begin{bmatrix} 0 \\ 0 \end{bmatrix}$$

(S89)

As before, the sign of the eigenvector is not changed.

So, the final eigenvectors are:

$$\boldsymbol{V_b} = \frac{1}{\sqrt{5}} \begin{bmatrix} 2 & -1 \\ 1 & 2 \end{bmatrix} = \begin{bmatrix} 0.8944 & -0.4472 \\ 0.4472 & 0.8944 \end{bmatrix} \quad \text{(S90)}$$

Transformation. The coordinates of molecule *B* can be defined into the canonical basis of the PC space as $\boldsymbol{B'}$ (PCA scores).

$$\begin{aligned} \boldsymbol{B'} &= \boldsymbol{BV_b} \\ &= \begin{bmatrix} -1 & -0.5 \\ 1 & 0.5 \end{bmatrix} \frac{1}{\sqrt{5}} \begin{bmatrix} 2 & -1 \\ 1 & 2 \end{bmatrix} \\ &= \frac{1}{\sqrt{5}} \begin{bmatrix} -2 - 0.5 & 1 - 1 \\ 2 + 0.5 & -1 + 1 \end{bmatrix} \\ &= \frac{1}{\sqrt{5}} \begin{bmatrix} -2.5 & 0 \\ 2.5 & 0 \end{bmatrix} \\ &= \begin{bmatrix} -1.118 & 0 \\ 1.118 & 0 \end{bmatrix} \end{aligned}$$

(S91)

Reference points.

$ref_1 = [0, 0, 0, 0, 0, 0]$

$ref_2 = [1.118, 0, 0, 0, 0, 0]$

$ref_3 = [0, 1, 0, 0, 0, 0]$

$ref_4 = [0, 0, 1, 0, 0, 0]$

$ref_5 = [0, 0, 0, 1, 0, 0]$

$ref_6 = [0, 0, 0, 0, 1, 0]$

$ref_7 = [0, 0, 0, 0, 0, 1]$

$$\begin{bmatrix} 0 & 0 & 0 & 0 & 0 & 0 \\ 1.118 & 0 & 0 & 0 & 0 & 0 \\ 0 & 1 & 0 & 0 & 0 & 0 \\ 0 & 0 & 1 & 0 & 0 & 0 \\ 0 & 0 & 0 & 1 & 0 & 0 \\ 0 & 0 & 0 & 0 & 1 & 0 \\ 0 & 0 & 0 & 0 & 0 & 1 \end{bmatrix} \quad \text{(S92)}$$

Distances.

$d_{ref1} = (1.118, 1.118)$

$$d_{ref2} = (2.236, 0)$$
$$d_{ref3} = (1.5, 1.5)$$
$$d_{ref4} = (1.5, 1.5)$$
$$d_{ref5} = (1.5, 1.5)$$
$$d_{ref6} = (1.5, 1.5)$$
$$d_{ref7} = (1.5, 1.5)$$

Moments.

$$moments_1 = (1.118, 0, 0)$$
$$moments_2 = (1.118, 1.118, 0)$$
$$moments_3 = (1.5, 0, 0)$$
$$moments_4 = (1.5, 0, 0)$$
$$moments_5 = (1.5, 0, 0)$$
$$moments_6 = (1.5, 0, 0)$$
$$moments_7 = (1.5, 0, 0)$$

Fingerprint.

$$\boldsymbol{f_B} = [1.118, 0, 0, 1.118, 1.118, 0, 1.5, 0, 0, 1.5, 0, 0, 1.5, 0, 0, 1.5, 0, 0, 1.5, 0, 0]$$

S.2.3. Similarity

Manhattan distance. The Manhattan distance is computed as

$$\begin{aligned} d_M &= |\boldsymbol{f_A} - \boldsymbol{f_B}| \\ &= [1.412, 0, 0, 1.412, 1.412, 0, 1.732, 0, 0, 1.732, 0, 0, 1.732, 0, 0, 1.732, 0, 0, 1.732, 0, 0] \\ &\quad - [1.118, 0, 0, 1.118, 1.118, 0, 1.5, 0, 0, 1.5, 0, 0, 1.5, 0, 0, 1.5, 0, 0, 1.5, 0, 0]| \end{aligned} \tag{S93}$$

There are only two different comparisons that we need to compute the partial score:

$$|1.414 - 1.118| = 0.296 \tag{S94}$$

$$|1.732 - 1.500| = 0.232 \tag{S95}$$

$$Partial\ score = \frac{1}{21}(d_M) = \frac{1}{21}(0.296 \cdot 3 + 0.232 \cdot 5) = 0.098 \tag{S96}$$

Similarity:

$$S = \frac{1}{1+0.098} = 0.911 \tag{S97}$$

To reproduce these results, see https://doi.org/10.5281/zenodo.14631654.

S.3. Chirality Detection Methods and Symmetry

Methods like Chiral Shape Recognition (CSR)[2] and USR:OptIso[3] utilize cross or triple products, generated from vectors between designated reference points, to select a further reference point in a chirality-dependent manner, i.e., to obtain a reference point that is not invariant under reflection, and, hence, construct different fingerprints for enantiomers, i.e., molecules which are non-superimposable mirror images of each other. While these methods succeed in distinguishing enantiomers, they are all affected by the inability to assign unambiguous reference points in cases where symmetry creates equally valid sets of reference points. One such example is presented in Figure S1.

Although the two methods are different, they both have a cross-product as the root of the chirality-detection method (the triple product is computed by taking the cross product of two vectors and then taking the dot product of the resultant vector with a third vector).

Therefore, here we consider the CSR method, but the same considerations apply to USR:OptIso. In addition to defining the geometrical center *(ctd*, light-blue sphere in Figure S1), the CSR method identifies the atom furthest from *ctd (ftc)* and then the atom furthest from *ftc (ftf)*. Given the symmetric molecular geometry of cyanoform, both *ftc* and *ftf* will be located on one of the three terminal nitrogen atoms. As graphically illustrated in Figure S1, the result of the cross product ($\boldsymbol{v}_3$, blue vector) between the vector $\boldsymbol{v}_1 = ftc - ctd$ (red vector) and $\boldsymbol{v}_2 = ftf - ctd$ (green vector) depends on which pair of nitrogen atoms are chosen as *ftc* and *ftf*. Since these nitrogen atoms are all equidistant from the geometrical center *(ctd),* the choice risks to be determined by the algorithm's implementation details. For example, if the algorithm prioritizes the first (or last) atom in the list that meets the selection criteria, the order in which atoms are listed also affects the selection process.

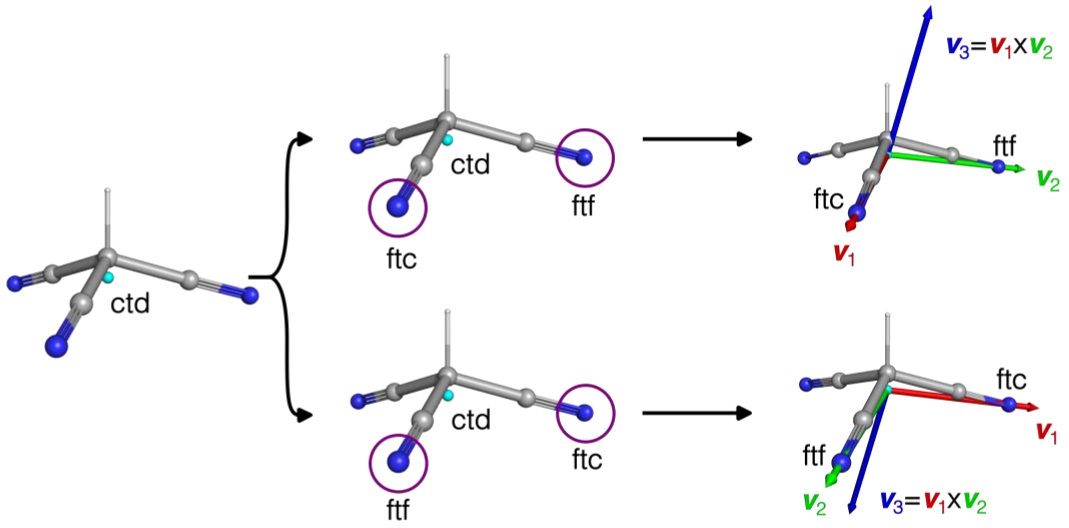

Figure S1. Molecular symmetry causes ambiguity in assignment of second (*ftc*) and third (*ftf*) reference point which causes ambiguity in the assignment of the fourth reference point.

As a result, two copies of the same molecule that differ only by the order in which the atoms are listed may return a similarity score widely different from the unit. Similarly, minor coordinate difference and numerical precision may as well result in overestimation of shape dissimilarity.

S.4. Computational Details

The details to reproduce the figures and the similarity scores presented in the main paper are available in Zenodo at https://doi.org/10.5281/zenodo.14631654, while our implementation of the HSR software is available at https://github.com/denoptim-project/HSR. The following sections provide additional details and the locations of the specific data and scripts for every figure in the main paper.

S.4.1. USR Outline

The molecule used in Figure 1 of the main paper has been obtained from Ref. 4.

The molecular structure and a script to reproduce the figure are available in the *USR_outline* directory at https://doi.org/10.5281/zenodo.14631654.

S.4.2. HSR Reference Points

The molecule used in Figure 2 of the main paper has been obtained from Ref. 4.

For the molecular structure and scripts used to generate the figure see the *HSR_reference_points* directory at https://doi.org/10.5281/zenodo.14631654.

S.4.3. HSR Chirality Method

The molecule used in Figure 3 of the main paper has been obtained as the molecules described in S.4.7.

For the molecular structures and scripts used to generate the figure see the *HSR_chirality_method* directory at https://doi.org/10.5281/zenodo.14631654.

S.4.4. DUD-E Experiments

For comprehensive details on the implementation of the HSR script designed to emulate the USR, USRCAT, and ElectroShape methods, see https://doi.org/10.5281/zenodo.14631654. The directory includes scripts to run the DUD-E experiments with the similarity measures implemented in the RDKit[5] and ODDT[6] software packages.

A python script is available in the *DUD-E* directory at https://doi.org/10.5281/zenodo.14631654. and includes three emulation methods, named *pseudo_usr*, *pseudo_usrcat*, and *pseudo_electroshape*.

In all cases the similarity measure differs from the original implementations in the selection of reference points as it utilizes Principal Component Analysis (PCA) instead of the original selection of *ctc*, *ftc*, and *ftf* atoms based on inter-atomic distances.

The atom type definitions in *pseudo_usrcat* are based on the matching SMARTS queries equal to those defined in the RDKit's implementation of the USRCAT method.

In the implementation of *pseudo_electroshape*, the partial charges are calculated using the ODDT package to obtain the same values used in the ODDT's ElectroShape method for a better comparison.

The three methods have been evaluated based on their ability to compute enrichment factors at various percentages: 0.25%, 0.5% (which is reported in the main paper), 1%, 2%, 3%, and 5%. (see Table S1, Table S2, Table S3 in the Computational Data Section)

S.4.5. Continuity

The molecules shown in Figure 4 of the main paper were chosen to reflect the experience described in Ref. 3. The 3D structures were obtained from a conformational search in Spartan20[7] using the Merck molecular force field[8] and selecting the conformers displayed in Ref. 3, which are those that lead to discontinuity issue described in the main paper.

For the molecular structures and the script to generate the results see the *Continuity* directory at https://doi.org/10.5281/zenodo.14631654.

S.4.6. Inorganics

The structures in Figure 5A of the main paper were obtained from Ref. 9, while the other structures were obtained from the Crystallography Open Database (COD).[10,11] Specifically, structures in Figure 5B were obtained from Ref. 12, and structures in Figure 5C were obtained from Ref. 13.

For the molecular structures and scripts used to generate the figure and results see the *Inorganic* directory at https://doi.org/10.5281/zenodo.14631654.

S.4.7. Chirality

All structures shown in Table 2 of the main paper were obtained by manually constructing one of the enantiomers, perform a conformational search with the Merck molecular force field[8] as implemented in Spartan20[7] to select the lowest-energy conformer. The enantiomer was generated by mirroring across a plane. This method ensures that chirality is the only difference between each enantiomer pair, aside from numerical noise. Consequently, any variations in similarity scores between these pairs reflects exclusively the different chirality.

The structure shown in Figure 6 of the main paper was obtained from the Crystallography Open Database (COD).[10,11] Specifically from Ref. 14. Its enantiomeric counterpart has been obtained by mirroring the first structure as described above.

For the molecular structures and the scripts to generate the results see the *Chirality* directory at https://doi.org/10.5281/zenodo.14631654. This includes also an in-house implementation of the CSR and the USR:OptIso methods.

S.4.8. Features

The molecular structures shown in Figure 7 of the main paper were constructed manually with Spartan'20.[7] These geometries are not optimized to ensure that the conformation remains as close as possible to the idealized geometry generated by hand. This is intended to purge the results from any effect due to molecular modelling of the geometries.

For the molecular structures and scripts to generate the results and the figure see the *Features* directory at https://doi.org/10.5281/zenodo.14631654.

S.4.9. Position Dependency

The molecule shown in Figure 8 of the main paper was obtained from Ref. 15.

For the molecular structures and the scripts to generate the results and the figure see the *Position_dependency* directory at https://doi.org/10.5281/zenodo.14631654.

S.5. Computational Data

S.5.1. DUD-E

Table S1. DUD-E Enrichment experiments for RDKit´s implementation of USR and USRCAT similarity measures.

Enrichment factor percentage (%)	RDKit	
	USR	USRCAT
0.25	7.09	14.43
0.5	5.44	10.95
1.0	4.16	7.92
2.0	3.23	5.59
3.0	2.83	4.55
5.0	2.40	3.50

Table S2. DUD-E Enrichment results for ODDT´s implementation of USR, USRCAT and ElectroShape similarity measures.

Enrichment factor percentage (%)	ODDT		
	USR	USRCAT	ElectroShape
0.25	5.82	13.18	13.09
0.5	4.52	9.81	10.27
1.0	3.55	6.94	7.71
2.0	2.84	4.82	5.57
3.0	2.52	3.89	4.55
5.0	2.18	2.99	3.51

Table S3. DUD-E Enrichment results for HSR's emulation of USR, USRCAT, and ElectroShape similarity measures.

Enrichment factor percentage (%)	HSR		
	Pseudo_USR	Pseudo_USRCAT	Pseudo_ElectroShape
0.25	9.09	15.02	11.99
0.5	6.84	11.58	9.21
1.0	5.05	8.48	6.79
2.0	3.74	6.00	4.96
3.0	3.16	4.88	4.13
5.0	2.58	3.76	3.28

S.5.2. Runtimes

Table S4 presents the runtimes of the DUD-E experiments, showing that RDKit is the fastest, likely due to its core implementation in C, which outperforms the native Python implementation in HSR and ODDT. Interestingly, despite its higher computational complexity, HSR achieves runtimes comparable to ODDT, suggesting a more efficient implementation than ODDT.

Table S4. DUD-E Runtimes for Selected Implementations of Similarity Methods.

Similarity Method	Runtime (minutes)		
	RDKit	ODDT	HSR
USR	40	62	47[a]
USRCAT	48	111	115[b]
ElectroShape	n.a.	70	70[c]

[a]Single 3D *hypershape*. [b]Combination of five 3D *hypershapes*. [c]Single 4D *hypershape*.

HSR runtimes are expected to grow with the number of atomic features considered in addition to the Cartesian coordinates. In fact, computing the fingerprint of cyclopentadienyliron tricarbonyl (17 atoms) takes on average over 1000 repetitions 1.74 ms in 3D, with an increase of runtime of +15% in 4D (2.01 ms), +30% in 5D (2.27 ms), and +40% in 6D (2.44 ms). All computations were conducted on an 8-core MacBook Pro with an Apple M1 Pro chip.

To further investigate HSR runtimes—specifically, the time required to generate fingerprints and perform similarity comparisons—we conducted speed tests using randomly selected molecules from the ZINC20 database.[16] One million 3D molecules were randomly sampled, and their default HSR fingerprints were computed while recording the time taken. Subsequently, a single molecule was randomly selected and compared against all others, simulating a typical use case in which the method is employed to retrieve the most similar candidates from a large molecular set.

This experiment was repeated 10 times, each time selecting a different set of random molecules. On average, fingerprint generation took approximately 3.5 milliseconds per molecule, with individual runtimes ranging from 1.5 to 150 milliseconds. The average time required to compute one milion pairwise similarity comparisons was 14 seconds.

Validation of In-House USR Implementation

All USR results reported in the main paper were produced from an in-house implementation of the USR method (see https://doi.org/10.5281/zenodo.14631654) to allow for the examination of intermediate results, such as the locations of reference points. To validate our implementation, we compared the results against those produced with the RDKit version of the USR method. Results

from both implementations are presented side by side for the Continuity and Inorganics experiments in Table **S5** and Table **S6**.

S.5.2.1. Continuity

Table S5. Validation of in-house USR implementation: comparative analysis of similarity scores derived from the in-house implementation versus RDKit's USR implementation for the Continuity experiments.

	Conformers A	Conformers B
(in-house) USR	0.7829	0.8676
(RDKit) USR	0.7829	0.8676

S.5.2.2. Inorganics

Table S6. Validation of in-house USR implementation: comparative analysis of similarity scores derived from the in-house implementation versus RDKit's USR implementation for the Inorganics experiments.

	1-linkage_isomerism	2-fac_mer_isomerism	3-cis_trans_isomerism
(in-house) USR	0.9961	0.9927	0.7117
(RDKit) USR	0.9961	0.9927	0.7117
(RDKit) USRCAT	0.5265	0.9125	0.3890

S.5.2.3. USRCAT's Connectivity Dependency

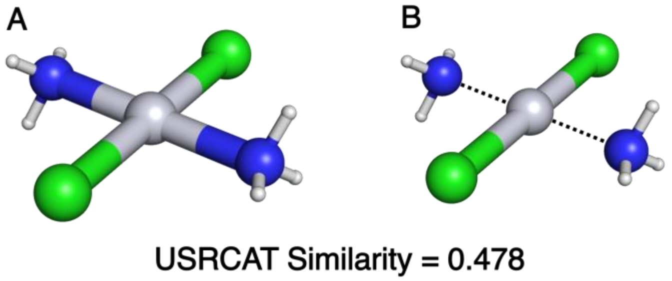

Figure S2. RDKit's USRCAT comparison of the same arrangement of atoms associated with different connectivity tables. (A) The dative bonds between Pt and NH_3 are described as single bonds. (B) The dative bonds are removed. Structures generated manually with Spartan20.[7]

For the molecular structures and scripts used to generate the figure and results see the *Inorganic* directory at https://doi.org/10.5281/zenodo.14631654.

S.6. References

(1) Strang, G. *Linear Algebra and Its Applications*, 4th ed.; Brooks/Cole: Belmont, CA, 2006.

(2) Armstrong, M. S.; Morris, G. M.; Finn, P. W.; Sharma, R.; Richards, W. G. Molecular Similarity Including Chirality. *J. Mol. Graph. Model.* **2009**, *28*, 368–370. https://doi.org/10.1016/j.jmgm.2009.09.002.

(3) Zhou, T.; Lafleur, K.; Caflisch, A. Complementing Ultrafast Shape Recognition with an Optical Isomerism Descriptor. *J. Mol. Graph. Model.* **2010**, *29*, 443–449. https://doi.org/10.1016/j.jmgm.2010.08.007.

(4) Fun, H.-K.; Ng, S.-L.; Li, Z.; Xu, J.-H. (2,5-Dichloro-4-Hydroxy-phen-yl)(Phen-yl)-methanone. *Acta Crystallogr. Sect. E Struct. Rep. Online* **2007**, *63*, o3121–o3121. https://doi.org/10.1107/S1600536807026864.

(5) RDKit: Open-Source Cheminformatics, 2023_09_3 (Q3 2023) Release, 2023. https://doi.org/10.5281/zenodo.10275225.

(6) Wójcikowski, M.; Zielenkiewicz, P.; Siedlecki, P. Open Drug Discovery Toolkit (ODDT): A New Open-Source Player in the Drug Discovery Field. *J. Cheminformatics* **2015**, *7*, 26. https://doi.org/10.1186/s13321-015-0078-2.

(7) Wavefunction Inc. Spartan'20, 2020.

(8) Halgren, T. A. Merck Molecular Force Field. I. Basis, Form, Scope, Parameterization, and Performance of MMFF94. *J. Comput. Chem.* **1996**, *17*, 490–519. https://doi.org/10.1002/(SICI)1096-987X(199604)17:5/6<490::AID-JCC1>3.0.CO;2-P.

(9) Sanz García, J.; Talotta, F.; Alary, F.; Dixon, I. M.; Heully, J.-L.; Boggio-Pasqua, M. A Theoretical Study of the N to O Linkage Photoisomerization Efficiency in a Series of Ruthenium Mononitrosyl Complexes. *Molecules* **2017**, *22*, 1667. https://doi.org/10.3390/molecules22101667.

(10) (IUCr) Crystallography Open Database – an open-access collection of crystal structures https://journals.iucr.org/j/issues/2009/04/00/kk5039/index.html.

(11) Gražulis, S.; Daškevič, A.; Merkys, A.; Chateigner, D.; Lutterotti, L.; Quirós, M.; Serebryanaya, N. R.; Moeck, P.; Downs, R. T.; Le Bail, A. Crystallography Open Database (COD): An Open-Access Collection of Crystal Structures and Platform for World-Wide Collaboration. *Nucleic Acids Res.* **2012**, *40*, D420–D427. https://doi.org/10.1093/nar/gkr900.

(12) Frances-Monerris, A.; Magra, K.; Darari, M.; Cebrián, C.; Beley, M.; Domenichini, E.; Haacke, S.; Pastore, M.; Assfeld, X.; Gros, P. C.; Monari, A. Synthesis and Computational Study of a Pyridylcarbene Fe(II) Complex: Unexpected Effects of Fac/Mer Isomerism in Metal-to-Ligand Triplet Potential Energy Surfaces. *Inorg. Chem.* **2018**, *57*, 10431–10441. https://doi.org/10.1021/acs.inorgchem.8b01695.

(13) Rivera, C.; Bacilio-Beltrán, H. A.; Puebla-Pérez, A. M.; Rangel-Salas, I. I.; Alvarado-Rodríguez, J. G.; Flores-Moreno, R.; Velázquez-Juárez, G.; Peregrina-Lucano, A. A.; Becerra-Martínez, E.; Valdez-Ruvalcaba, J.; Rubio, J. E.; Cortés-Llamas, S. A. Cis and Trans Platinum(II) N-Heterocyclic Carbene Isomers: Synthesis, Characterization and Biological Activity. *New J. Chem.* **2022**, *46*, 14221–14226. https://doi.org/10.1039/D2NJ02508F.

(14) Wendt, M.; Warzok, U.; Näther, C.; Leusen, J. van; Kögerler, P.; Schalley, C. A.; Bensch, W. Catalysis of "Outer-Phase" Oxygen Atom Exchange Reactions by Encapsulated "Inner-Phase" Water in {$V_{15}Sb_6$}-Type Polyoxovanadates. *Chem. Sci.* **2016**, *7*, 2684–2694. https://doi.org/10.1039/C5SC04571A.

(15) Lee, D.; Newman, S. G.; Taylor, M. S. Boron-Catalyzed Direct Aldol Reactions of Pyruvic Acids. *Org. Lett.* **2009**, *11*, 5486–5489. https://doi.org/10.1021/ol902322r.

(16) Irwin, J. J.; Tang, K. G.; Young, J.; Dandarchuluun, C.; Wong, B. R.; Khurelbaatar, M.; Moroz, Y. S.; Mayfield, J.; Sayle, R. A. ZINC20—A Free Ultralarge-Scale Chemical Database for Ligand Discovery. *J. Chem. Inf. Model.* **2020**, *60*, 6065–6073. https://doi.org/10.1021/acs.jcim.0c00675.
